# One class classification for the detection of β2 adrenergic receptor agonists using single-ligand dynamic interaction data

**DOI:** 10.1186/s13321-022-00654-z

**Published:** 2022-10-29

**Authors:** Luca Chiesa, Esther Kellenberger

**Affiliations:** Laboratoire d’innovation Thérapeutique, Faculté de Pharmacie, UMR7200 CNRS Université de Strasbourg, 67400 Illkirch, France

**Keywords:** Machine learning, Virtual screening, Molecular dynamics, ADRB2, GPCRs, One class classification

## Abstract

G protein-coupled receptors are involved in many biological processes, relaying the extracellular signal inside the cell. Signaling is regulated by the interactions between receptors and their ligands, it can be stimulated by agonists, or inhibited by antagonists or inverse agonists. The development of a new drug targeting a member of this family requires to take into account the pharmacological profile of the designed ligands in order to elicit the desired response. The structure-based virtual screening of chemical libraries may prioritize a specific class of ligands by combining docking results and ligand binding information provided by crystallographic structures. The performance of the method depends on the relevance of the structural data, in particular the conformation of the targeted site, the binding mode of the reference ligand, and the approach used to compare the interactions formed by the docked ligand with those formed by the reference ligand in the crystallographic structure. Here, we propose a new method based on the conformational dynamics of a single protein–ligand reference complex to improve the biased selection of ligands with specific pharmacological properties in a structure-based virtual screening exercise. Interactions patterns between a reference agonist and the receptor, here exemplified on the β2 adrenergic receptor, were extracted from molecular dynamics simulations of the agonist/receptor complex and encoded in graphs used to train a one-class machine learning classifier. Different conditions were tested: low to high affinity agonists, varying simulation duration, considering or ignoring hydrophobic contacts, and tuning of the classifier parametrization. The best models applied to post-process raw data from retrospective virtual screening obtained by docking of test libraries effectively filtered out irrelevant poses, discarding inactive and non-agonist ligands while identifying agonists. Taken together, our results suggest that consistency of the binding mode during the simulation is a key to the success of the method.

**Supplementary Information:**

The online version contains supplementary material available at 10.1186/s13321-022-00654-z.

## Introduction

G protein-coupled receptors (GPCRs) constitute the largest family of membrane proteins in humans. GPCRs mediate the cellular response to outside stimuli by initiating specific signaling transduction pathways [1]. As key player in many physiological processes, GPCRs have been largely exploited as drug targets [2]. The pharmacological profile of a GPCR ligand, whether endogenous or xenobiotic, is characterized according to the response it induces in cell [3]: agonist triggers signaling, unlike antagonist and inverse agonist (henceforth referred to as antagonist too, for simplicity). The computer-assisted discovery of GPCR ligands for therapeutic purposes therefore requires the development of predictive models of the pharmacological profile. Machine learning well suits this purpose by extracting complex relationship from experimental data and classification algorithms are typically adapted to distinguish agonists from antagonists [4, 5]. However, their application is limited to well-studied protein targets with many known agonists and antagonists. Methods based on the three-dimensional structure of the protein–ligand complex can circumvent this limitation. Since the resolution of the first structure of a GPCR bound to an antagonist in 2007 [6] and coupled to G-protein in 2011 [7], the continuous elucidation of new protein–ligand structures has led to a golden age for structure based drug design projects [8–10]. The abundant structural information also highlighted the GPCR’s structural plasticity. GPCRs adopt a wide spectrum of conformational states, ranging from inactive to active ones.

In the search for novel and original ligands of GPCRs, ligand/protein docking accounts for the most common tool for virtual screening of chemical libraries [11]. Docking predicts the binding mode and scores the solutions. The score can be interpreted in terms of ligand binding, but not ligand function.

While agonist ligands are known to have a higher binding affinity for the active state, and antagonists for the inactive state[12], the structures of the binding pocket in both states tend to be very similar [11]. Agonist ligands have thus been identified by virtual screening using an inactive state of the receptor [13–17] and antagonists using an active state [18]. The analysis of the binding mode, by comparison with reference crystallographic structures, better accounts for the pharmacophoric specificity of agonists and antagonists [12, 19]. For example Kooistra et al. [12] investigated the use of experimental reference binding modes to functionally bias the screening against the β-adrenoreceptor family. Their retrospective analysis showed that it is possible to improve the retrieval of agonist or antagonist ligands from a test library containing active molecules and decoys by assessing the similarity between the binding mode generated by docking with the crystallographic binding mode, here by the comparison of interaction fingerprints (IFP). By considering the enrichment factor at 1% false positive rate, a reference agonist IFP increased the proportion of agonists among the true positives, and similarly, a reference antagonist IFP increased the proportion of antagonists. Despite the overall improvement different reference ligands, having different IFPs, yielded different hit lists. Even the binding mode of the same ligand in multiple crystallographic structures can vary [12, 20]. The changes in binding mode can be explained by the inherent flexibility of GPCRs [3] or by different conditions of crystallization (e.g., thermostabilizing mutations, coupled nanobodies) [21]. Selecting a reference structure containing the relevant interaction patterns plays a key role in virtual screening [12]. The best reference can be identified by retrospective virtual screening experiments, yet such approach requires data from known agonists, antagonists, and inactive molecules [22].

The β2 adrenergic receptor (ADRB2) has been the first receptor crystalized bound to a ligand and coupled to G-protein. At the time of writing the structure of the receptor has been elucidated by thirty-seven crystallographic structures. ADRB2 has been thoroughly studied due to the crucial role of both its agonist and antagonists in drug discovery. The receptor is present mostly in lung tissues, where its signaling regulates smooth muscle relaxation and bronchodilation. Agonists are used to treat respiratory disorders[23] such as asthma[24] and chronic obstructive pulmonary disease (COPD)[25]. Antagonists, more commonly known as β-blockers, are used to treat cardiovascular diseases by targeting the β1 adrenergic receptors in heart tissues. The action of β-blockers on ADRB2 is mostly linked to undesirable side-effects [26]. Thanks to the large amount of information regarding both its structure and pharmacology, ADRB2 has been used in computational works as a model system [12, 22].

Key interactions for GPCR activation are inferred from the analysis of experimental structures of the receptor bound to different agonists, provided experimental structures are available for several agonist/GPCR complexes [27].Considering a single agonist-GPCR structure, molecular dynamics (MD) calculations can evaluate intermolecular interactions, based on the principle that the key interactions are better conserved during simulation [27, 28]. Here, we consider a single agonist-GPCR structure and propose to combine MD simulation and machine learning to bias the search towards agonist ligands by virtual screening of chemical libraries using docking. The proof of concept was performed on ADRB2, for which we constructed two suitable test sets: A library containing ADRB2-targeting drugs or clinical candidates with a well characterized pharmacological profile, and a library composed of virtual hits selected by docking into ADRB2 orthosteric site and validated/invalidated for agonist activity [29]. A one class support vector machine (OCSVM) [30] model was trained on the ADRB2-agonist interactions extracted from a single MD trajectory and represented by graphs, allowing a binding mode definition based on interactions conservation. The classifier was used to filter ligand–ADRB2 docking generated poses. The ability of each classifier to distinguish between agonist and not agonist was determined considering the fraction of true agonists between the selected molecules (precision) and the fraction of true agonists selected from the test dataset (recall). The effect of simulation length, interaction types, combinations of multiple references, and training method were taken into consideration by training multiple classifiers, with different combinations of parameters. The results of the analysis can be used as guidelines for the training of new models targeting different GPCRs. The proposed methodology can be applied to find new agonists for targets with a limited number of known reference ligands.

## Results and discussion

### Introduction

GPCRs classically mediate responses to agonist binding by coupling to effector, typically a G-protein. The activation mechanism and the resulting signaling are intimately connected to the conformation of the receptor, which itself is stabilized or induced by the interactions formed with the agonist. The process is well characterized for ABDR2 activation by the endogenous agonists epinephrine and norepinephrine. For example, hydrogen bonding to Ser207 has been proposed as key determinant of agonist binding [31]. In the present work, we consider the relationships between structure and function to be unknown and assess whether a machine learning model trained on the structural information of a single ligand is able to determine key agonist-related interactions, assuming that the patterns of interactions that are the most conserved during MD simulation of the receptor-agonist complex are also the most relevant for signaling. The machine learning model is applied to assess the compatibility of the interaction patterns predicted by docking for any ligand of the receptor with the common definition determined from the training data on the reference agonist. A ligand evaluated positively thus potentially exhibits a mode of binding similar to that of the reference agonist and is supposed to trigger the same pharmacological response.

### Model development

#### Reference agonists and MD simulations

**Fig. 1:**
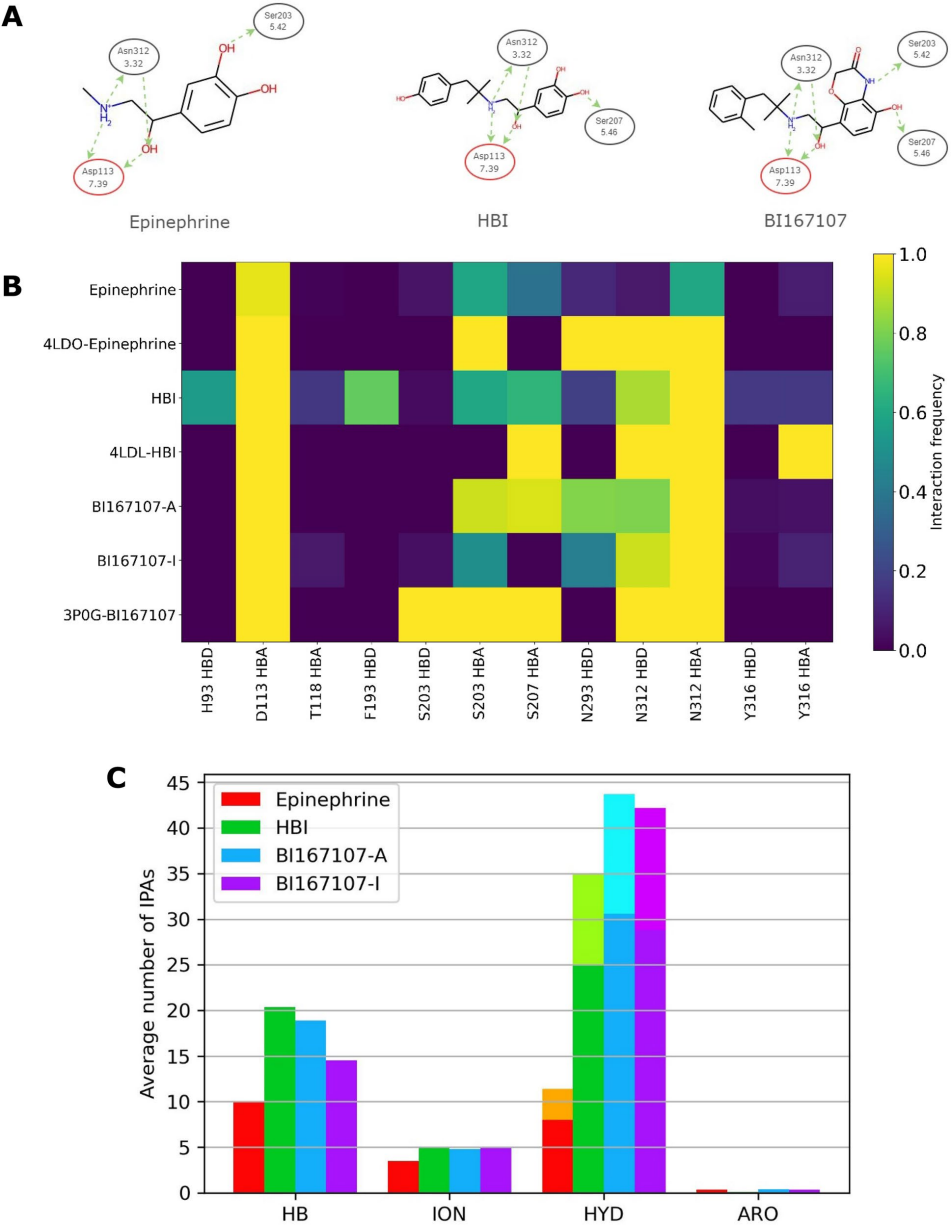
**A** Structure of the three reference agonists showing the hydrogen bonds observed in the crystallographic structures. The ADRB2 residues are identified by their sequence number and by Ballesteros-Weinstein notation. **B** IFPs with hydrogen bonds formed between the agonist and ABDR2 in the trajectory and crystallographic structures. HBA indicates interactions with the protein acting as the hydrogen bond acceptor, HBD indicates interactions with the protein acting as the hydrogen bond donor. Crystallographic structure IFPs are indicated by their PDBID. **C** Average distribution of the interaction pseudo-atoms types during the MD trajectories. *HB* hydrogen bonds, *ION* ionic interactions, *HYD* hydrophobic contacts, *ARO* pi-pi interactions. The lighter-coloured and legend-coloured parts of HYD bars corresponds to the default (Hyd) and stricter (Newhyd) definition of hydrophobic contacts, respectively

Three different agonists were selected for generating the training sets from published or publicly available molecular dynamics simulations [32, 33]: Epinephrine, hydroxybenzyl isoproterenol (HBI) and BI167107 (Fig. 1A). Epinephrine is a low affinity agonist of ABDR2 [34]. As a member of the endogenous neurotransmitters of catecholamine family, it contains the 1,2-dihydroxybenzene group (i.e., catechol) and a side-chain amine. The side-chain amine is part of a 2-aminoethanol group common to ADRB2 agonists. HBI is a high potency binder of ADRB2 [34]. It is a superstructure of epinephrine that features a phenol group at the end of the sidechain. BI167107 is an ultra-high affinity binder of ADRB2 [34]. Its structure shares with epinephrine and HBI the 2-aminoethanol group, but not the catechol which is replaced by a different functional group, with one of the two hydroxyl groups replaced by a 3-morpholinone like moiety.

The crystallographic structures of ADBR2 suggest, for the three agonists, a strong anchoring of their 2-aminoethanol group by hydrogen bonding to residues in TM2 (Asp113) and TM7 (Asn312), as well as an interaction between the hydroxyl of catechol, or its substitute in BI167107, with a serine of TM5 (Fig. 1A) [7, 34]. The interactions formed between epinephrine and ADRB2 are not strictly conserved during the MD simulation (Fig. 1B and Additional file 1: Fig. S1). Moreover, new interactions are observed, including a hydrogen bond with Ser207. Binding mode variations are also marked in the HBI-ADRB2 MD trajectory, involving additional receptor residues in the interactions formed with the agonist. While epinephrine-ADBR2 and HBI-ADRB2 MD trajectories both describe the active state of the receptor, the MD trajectory of BI167107-ADRB2 [33] simulates the transition of the receptor from the active state to the inactive state, and therefore was split to distinguish the binding of BI167107 to the active state (BI167107-A) and to the inactive state (BI167107-I), thus giving two separated datasets. Importantly, hydrogen bonding to Ser207 is lost in BI167107-I. The shift from the active to the inactive state was observed despite the receptor being bound to BI167107, an agonist. The absence of an intracellular binding partner, which stabilizes the active state conformation, explains the transition[35]. The fully inactive state of the receptor was observed only after microseconds of simulation, a longer timescale than the one used in the other selected simulations (Table 1). The study of BI167107 bound to both the active and inactive state of the receptor was used to investigate the differences between models built using structural information close and far from the active state crystallographic structure.

**Table 1:** MD simulations used for the training dataset. The simulation length refers to a single replica and not the entire trajectory

Reference trajectory	Number of replicas	Number of frames	Simulation length (ns)	Interval between writing structures (ns)
Epinephrine	3	2500	500	0.200
HBI	3	2500	500	0.200
BI167107-A	1	1944	350	0.180
BI167107-I	1	7500	1350	0.180

A total of four training sets were used in this work. The Table 1 characterises the MD trajectories with the number of simulations, the simulations length, and the sampling frequency.

#### Interaction graph

For each of the four reference trajectories (epinephrine, HBI, BI167107-A and BI167107-I), the following interactions were detected between ADRB2 and the reference agonist using IChem [36]: Hydrogen bonds, ionic bonds, aromatic stacking and hydrophobic contacts. Each interaction is represented by a triplet of interactions pseudo-atoms (IPA), one placed on each of the two atoms involved in the interaction (ligand IPA and protein IPA), and a third one in the middle point of the segment they define (centre IPA). More details about IChem detection and encoding of interactions are given in the "Materials and methods" section.

The average number of IPAs is shown on Fig. 1C. Aromatic interactions are rare in the four reference trajectories. The other polar interactions, hydrogen and ionic bonds, represents together ca 13 (for epinephrine) to ca 25 IPAs (for HBI) per interactions pattern. The number of IPAs corresponding to hydrophobic contacts is equal to or greater than the number of IPAs corresponding to polar interactions, except in the epinephrine trajectory. Since the description of hydrophobic contacts does not rely on a consensual physical model, unlike that of polar interactions, we tested three conditions: the two IChem definitions of hydrophobic contacts and the exclusion of hydrophobic contacts. Hence, for each of the four reference trajectories, three sets of IPAs were considered: all interactions with default definition of hydrophobic contacts (Hyd), all interaction with stricter definition of hydrophobic contacts (Newhyd), and polar interactions only (Polar).

Interaction graphs (IGs) were generated from the detected interactions, with the IPAs representing the nodes. To include the largest number of topological information all nodes were connected to each other, forming a complete graph. The nodes were labelled according to the IChem interaction type and the represented position (protein, ligand, centre). The edges were described by a weighted adjacency matrix, the weights being equal to the Euclidean distances between the IPAs rounded to the nearest Å. The distance between two overlapping IPAs was set to 0.1, to establish an edge between the nodes.

Graph similarity assessment is a key problem in graph theory, which is why different types of graph kernels have been developed [37]. In this work graph similarity was evaluated using the shortest path kernel [38]. Graphs similarity is based on the common occurrence in the graphs of identical shortest paths between two nodes with the same label. In practice, a graph was converted into a fingerprint containing the frequency of each shortest path. Two fingerprints were compared by calculating the cosine similarity and the vector dot product, yielding a normalized and a non-normalized (NN) score, respectively. Thus, for each of the three interaction definitions (Hyd, NewHyd and Polar), both normalized and non-normalized similarity scores were calculated.

#### Model training

Each MD trajectory contains information on a single class. To process data from a single class it is necessary to use algorithms built to handle limited information. One class classification or outlier detection [39, 40] is a family of semi-supervised classification algorithms whose focus is not to determine the boundary between two classes, but it is to learn a common definition describing the training instances. Such algorithms have already been proposed in ligand-based drug discovery [41], detection of druggable protein pockets [42], and in other fields of chemistry [43] characterized by a low availability of experimental data. Multiple outlier detection algorithms are available based on different well known machine learning models: OCSVM[30], isolation forest[44], k-nearest neighbours (KNN).

The OCSVM [30] algorithm was used to find a criterion to define conditions for the inclusion of a new graph into the ensemble of interactions observed during the reference trajectory. OCSVM, like other support vector machine-based classification methods, defines a class using a support vector (SV). In the case of OCSVM the SV represents the hyperplane with the maximum distance from the origin in the transformed feature space.

Training the OCSVM algorithm requires the user to set the hyperparameter ν to a value between 0 and 1. Two key quantities correspond to ν: The upper bound for the fraction of training instances classified as outliers during training, and the lower bound for the fraction of instances defining the SV. Since the effective tuning of the ν value is not possible in the absence of an external dataset, we followed heuristic approaches focusing on removing outliers or estimating the percentage of outliers in the training set.

Outliers in the training set were identified using the average shortest path kernel similarity value to the KNN, with K corresponding to 3% of the total number of training instances [45]. The first approach called quick model selection 2 (QMS2) [46], removes from the training set both outliers and instances on the boundaries of the distribution. Since the new training set should contain only true inliers the fraction of classification errors during training (ν) is set to a small value of 0.01. The second approach uses the median absolute deviation (MAD) to determine the number of outliers in the training set. The upper bound to the fraction of training errors is set to be equal to the number of instances with a distance from the median KNN similarity larger than three times the MAD divided by the total number of instances in the training set.

Models using normalized kernels were trained using both QMS2 and MAD, while the models based on NN scores were trained using QMS2 only. In short, for each of the four reference trajectories (epinephrine, HBI, BI167107-A and BI167107-I), nine models were constructed, combining three different sets of interactions (HYD or NEWHYD or POLAR) and three parametrizations (QSM2 or MAD or NN).

### Method validation

#### Introduction

The thirty-six trained OCSVM models were used to screen two small chemical libraries to evaluate their ability in recognizing agonist ligands. One library contains known agonists and antagonists, while the second contains experimentally validated agonists and inactive molecules.

For each molecule in the libraries, the docking into the three crystallographic structures of ABDR2 issued ten poses each. The calculations were repeated for up to nine representative structures of the receptor agonist binding site. Site definition and clustering of epinephrine-ADRB2, HBI-ADRB2, and BI167017-ADRB trajectories are detailed in Materials and Methods. Each model was used for post-processing only on the docking poses generated using a protein structure extracted from the respective training set. If the docking calculations generated at least one binding mode selected by an OCSVM model, then the molecule is classified as an agonist. Therefore, the performances discussed below apply to the entire virtual screening process, not only the classifiers.

#### Validation with agonist/antagonist dataset

The dataset contains 19 agonists and 17 antagonists extracted from the literature (Additional file 1:Table S1). All 36 molecules have been largely studied and their pharmacology is well known. The dataset was also curated to contain compounds with different chemical structures. A total of 24 different scaffolds are represented in the dataset, four scaffolds are shared by multiple compounds. Benzene is the only common scaffold between agonists and antagonists. All the molecules present the 2-ethanolamine group, except the agonist dobutamine. The agonists all present a benzene ring with a functional group able to form hydrogen bonds, like the catechol group of epinephrine, except tulobuterol whose benzene ring is substituted with a chlorine atom.

**Fig. 2:**
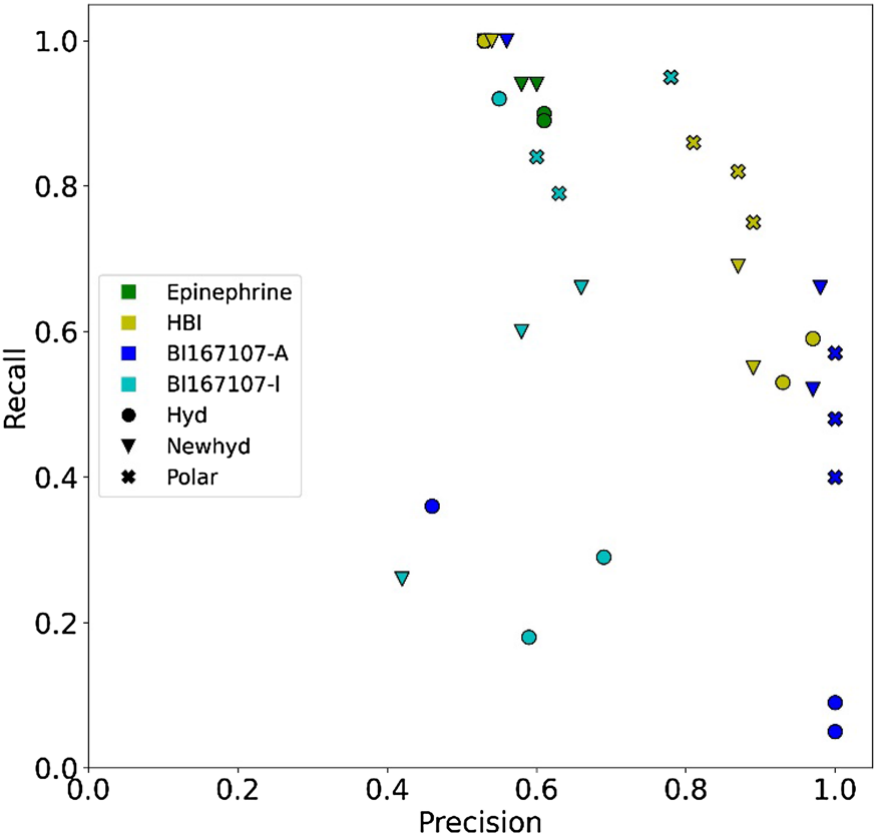
Average performance of OCSVM models obtained on the agonist/antagonist dataset from docking using the representative structures

The models performed differently depending on the definition of interactions, the normalization of the kernel, and the trajectory used as reference (Fig. 2 and Additional file 1:Tables S3, S4). However, it is possible to observe some general trends: Models based on epinephrine are all inefficient, predicting most to all molecules as agonists. HBI-based models correctly identified most of the true agonists and misclassified a limited number of antagonists. Training with the BI167107-A dataset generated models with a high precision, but with a tendency to discard many true agonists. Including hydrophobic contacts in the IGs was detrimental to NN models, causing all ligands to be classified as agonists. Likewise, the performances of models based on HBI and BI167107 overall improved if only polar interactions were included in the IGs.

The four best models for each reference trajectory are presented on Table 2. They were selected according to the average F1 measure calculated from docking poses using each representative structure, after discarding models which selected all ligands as agonists. For each model, the average performance over a set of representative conformations, selected from the respective training set, gives an estimate of the classifier behaviour. The four best HBI-based models retrieved three quarters or more of the agonists, while showing very few false positives. The best BI167107-based models discriminated between agonists and antagonists, provided they were trained from the portion of the trajectory simulating the active state of the receptor. BI167107-I-based models indeed incorrectly predicted many antagonists as agonists. The decrease of precision between models based on BI167107-A and BI167107-I is consistent with the vanishing of the key interaction with Ser207 upon deactivation of the receptor during the BI167107-ADRB2 simulation.

**Table 2:** Performance of the best four models obtained for each reference trajectory on the agonist/antagonist dataset from docking data obtained using the representative structures or the crystallographic structure

Reference trajectory	Model	Average			Crystallographic			Ensemble		
		Precision	Recall	Size	Precision	Recall	Size	Precision	Recall	Size
Epinephrine	Hyd–MAD	0.61	0.90	28/36	0.62	0.95	29/36	0.53	0.95	34/36
	Hyd–QMS2	0.61	0.89	28/36	0.58	0.95	31/36	0.55	0.95	33/36
	Newhyd–MAD	0.58	0.94	31/36	0.53	1.00	36/36	0.53	1.00	36/36
	Newhyd–QMS2	0.60	0.94	30/36	0.54	1.00	35/36	0.53	1.00	36/36
HBI	Polar–NN	0.87	0.82	18/36	0.94	0.79	16/36	0.73	1.00	26/36
	Polar–MAD	0.81	0.86	20/36	1.00	0.74	14/36	0.68	1.00	28/36
	Polar–QMS2	0.89	0.75	16/36	1.00	0.74	14/36	0.76	1.00	25/36
	Newhyd–MAD	0.87	0.69	15/36	1.00	0.68	13/36	0.72	0.95	25/36
BI167107-A	Polar–NN	1.00	0.57	11/36	1.00	0.89	17/36	1.00	0.84	16/36
	Polar–MAD	1.00	0.40	8/36	1.00	0.26	5/36	1.00	0.74	14/36
	Newhyd–QMS2	0.97	0.52	10/36	–	0.00	0/36	0.94	0.89	18/36
	Newhyd–MAD	0.98	0.66	13/36	–	0.00	0/36	0.94	0.89	18/36
BI167107-I	Polar–NN	0.78	0.95	23/36				0.79	1.00	24/36
	Polar–QMS2	0.60	0.84	27/36				0.59	0.89	29/36
	Polar–MAD	0.63	0.79	24/36				0.57	0.84	28/36
	Newhyd–MAD	0.66	0.66	19/36				0.67	0.84	24/36

Size refers to the fraction of molecules classified as agonists. Average refers to the mean of the values obtained for the representative structures considered individually. Ensemble refers to the value obtained by merging data from all representative structures. Models discarding all docking poses are indicated as –

For epinephrine and HBI reference trajectories, the results were comparable whether the considered docking poses use the crystallographic structure or a representative structure, on average. Similar findings were made for BI167107-A models based on polar interactions. The BI167107-A models including hydrophobic contacts did not select any molecule. An inappropriate conformation of the hydrophobic sub-pockets was assumed based on the two following evidences: The hydrophobic contacts between BI167107 and ADBR2 in the crystallographic structure and during the MD simulation differ (Additional file 1: Fig. S1); the binding site underwent significant structural changes during the simulation (Additional file 1: Fig. S2), with an average root mean squared deviation (RMSD) from the initial crystallographic structure above 3Å while the average pairwise RMSD between the frames is below 2.5Å (Additional file 1: Fig. S3, S4). For the sake of comparison, the RMSD values for epinephrine-ADBR2 and HBI-ADBR2 complexes are below 2.5Å (Additional file 1: Fig. S5, S6).

Since the performance of a model depends on the receptor’s structure, we also considered ensemble docking, by merging the docking poses obtained using all representative structures of the reference trajectory (Table 2). Overall, using all the representative structures together produced F1-measures comparable to or better than the average obtained by considering the representative structures separately. Increasing the number of evaluated poses improved recall, yet often at the expense of precision. The best improvement concerns BI167107-A high precision models, which showed a significant increase in recall (e.g. from 0.57 to 0.84 for the Polar-NN model) with no loss of precision (e.g., 1.00 for the Polar-NN model). Models characterized by a looser definition of agonist binding mode, such as HBI-based models and to a lesser extent BI167107-I, tend to include a larger number of false positives.

#### Validation with agonist/inactive dataset

**Fig. 3:**
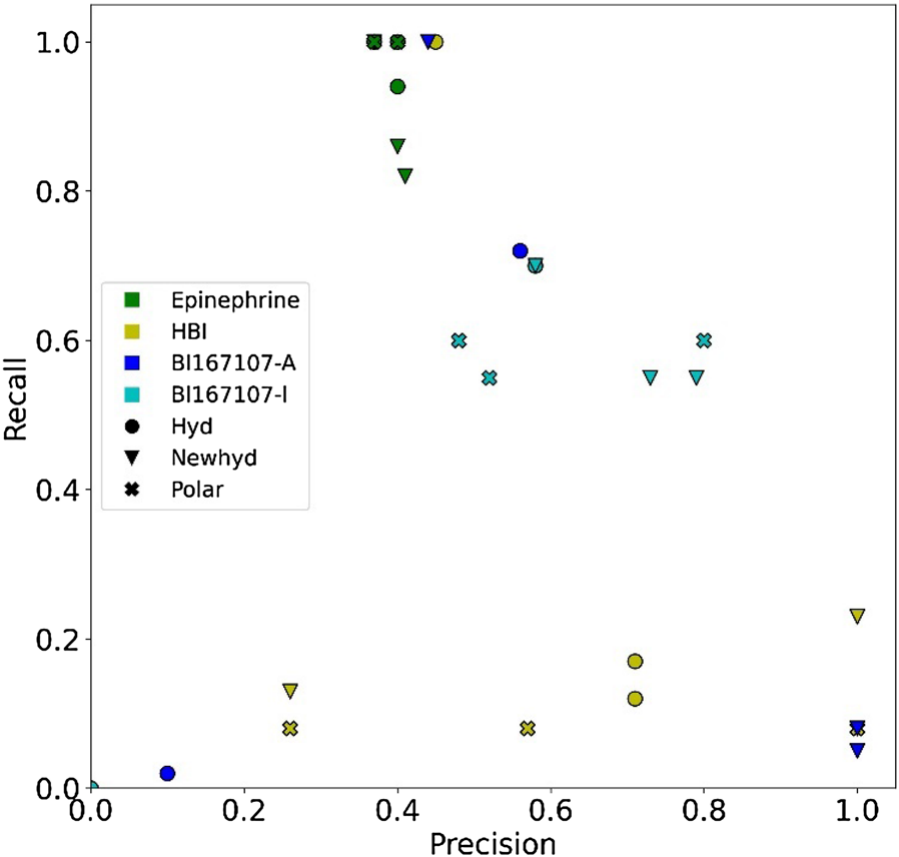
Average performance of OCSVM models on the agonist/inactive dataset from docking using the representative structures. Note that one Hyd and the three Polar models of BI167107-A are not visible since no molecules were predicted as active

To test the ability of the OCSVM models in correctly identifying inactive molecules as non-agonist a dataset of experimentally validated true agonists and true inactive molecules was selected from the literature [29]. The library consists of ten agonists and 17 inactive molecules. All the 27 molecules are virtual hits of a structure-based screening of the ZINC12 library. They represent 17 different scaffolds, including 3 which are specific for agonists and 14 are specific for inactive molecules. All active molecules and two inactive molecules contain the 2-aminoethanol group which is present in ADRB2 ligands and allows their anchoring to both TM3 and TM7. The agonists share a second common characteristic: all but one contain a halogenated aromatic ring instead of the catechol of epinephrine. The halogen atoms are assumed to interact with the subpocket formed by TM5, TM4, and TM3, for receptor activation upon agonist binding. With halogen interactions not being taken into account by IChem, the present dataset is highly challenging for the models. Overall, the performance of the models is worse on the agonist/inactive dataset than on the agonist/antagonist dataset (Fig. 3 and Additional file 1: Tables S5, S6). Again, epinephrine-based models are not predictive, classifying all or nearly all molecules of the agonist/inactive dataset as agonists.

The four best models for each reference trajectory are presented on Table 3. They were selected according to the average F1 measure calculated from docking poses using each representative structure, after discarding models which select all ligands as agonists. For each model, the average performance over a set of representative conformations, selected from the respective training set, gives an estimate of the classifier behaviour. The best HBI-based models retrieved on average a quarter of the agonists, yet they well classified the inactive molecules as non-agonists. The best BI16707-A models showed better recall, but at the expense of more false positives. For these two reference trajectories, models ignoring hydrophobic interactions missed even more agonists, likely due to the absence of any hydrogen bond to TM5 (Ser203 and Ser207) as a consequence of replacing the aromatic hydrogen bond donor of the canonical ADRB2 agonists with a halogen atom. However, these polar models successfully classified the inactive molecules as non-agonists. The BI167107-I-based best models show the best statistics on the agonist/inactive dataset, although they performed worse than on the agonist/antagonist set. Since the BI167107-I training set structures do not include the hydrogen bond between the reference agonist and ADRB2 Ser207, we can assume that the classifier is less impacted by the unusual binding mode of the test agonists which likely involves a halogen-protein or fluorine-protein interaction. Taken together, the results suggest that polar interactions patterns allow the classification of non-agonists, yet that the interaction with serine residues in TM5, which constitute a key pharmacophore, must be correctly appreciated by the model to identify agonists.

**Table 3:** Performance of the four best models obtained for each reference trajectory on the active/inactive dataset from docking data obtained using the representative structures

Reference trajectory	Model	Average			Ensemble		
		Precision	Recall	Size	Precision	Recall	Size
Epinephrine	Polar–MAD	0.40	1.00	25/27	0.37	1.00	27/27
	Hyd–QMS2	0.40	0.94	24/27	0.38	1.00	26/27
	Polar–QMS2	0.40	1.00	25/27	0.37	1.00	27/27
	Hyd–MAD	0.40	1.00	25/27			
HBI	Newhyd–QMS2	1.00	0.23	2/27	1.00	0.80	8/27
	Newhyd–MAD	1.00	0.23	2/27	1.00	0.70	7/27
	Hyd–NN	0.45	1.00	22/27	0.40	1.00	25/27
	Newhyd–NN	0.40	1.00	25/27	0.38	1.00	26/27
BI167107-A	Hyd–NN	0.56	0.72	13/27	0.56	1.00	18/27
	Newhyd–NN	0.44	1.00	23/27	0.42	1.00	24/27
	Newhyd–MAD	1.00	0.08	1/27	1.00	0.30	3/27
	Newhyd–QMS2	1.00	0.05	1/27	1.00	0.20	2/27
BI167107-I	Polar–NN	0.80	0.60	8/27	0.75	0.60	8/27
	Newhyd–NN	0.58	0.70	12/27	0.62	0.80	13/27
	Hyd–NN	0.58	0.70	12/27	0.62	0.80	13/27
	Newhyd–MAD	0.79	0.55	7/27	0.78	0.70	9/27

Size refers to the fraction of molecules classified as agonists. Average refers to the mean of the values obtained for the representative structures considered individually. Ensemble refers to the value obtained by merging data from all representative structures

As for the agonist/antagonist dataset the performance on the ensemble docking results was also considered. An important effect was observed for hydrophobic NN models, which under these conditions tend to select all molecules. By contrast, significant increase in recall with no loss in precision was observed for the best models based on HBI and BI167170-A. The better sampling of ligand bound conformations generated more poses containing hydrophobic contacts similar to the reference.

### Importance of sampling in characterizing the binding mode

#### Introduction

As for all machine learning models the characteristics of the training dataset reflect on the quality of the predictions. We questioned two aspects of binding mode sampling: the length of the MD simulation and the relevance of combining information from multiple references. Their effect on the classifiers’ performances on the antagonist/agonist dataset is discussed below.

#### Molecular dynamics simulation length

We repeated the OCSVM training using only the first 25%, 50%, and 75% of the frames of the MD simulations of epinephrine-ADRB2 and HBI-ABDR2 trajectories. These two trajectories were generated using the same experimental conditions. Both are formed by three different replicas, each one formed by 2500 frames, for a total of 7500 frames. Therefore, since they are the longest simulations and the only ones formed by more than a single replica, they were used to study the effect of the number of replicas and simulation length on the classifiers’ performances.

**Fig. 4:**
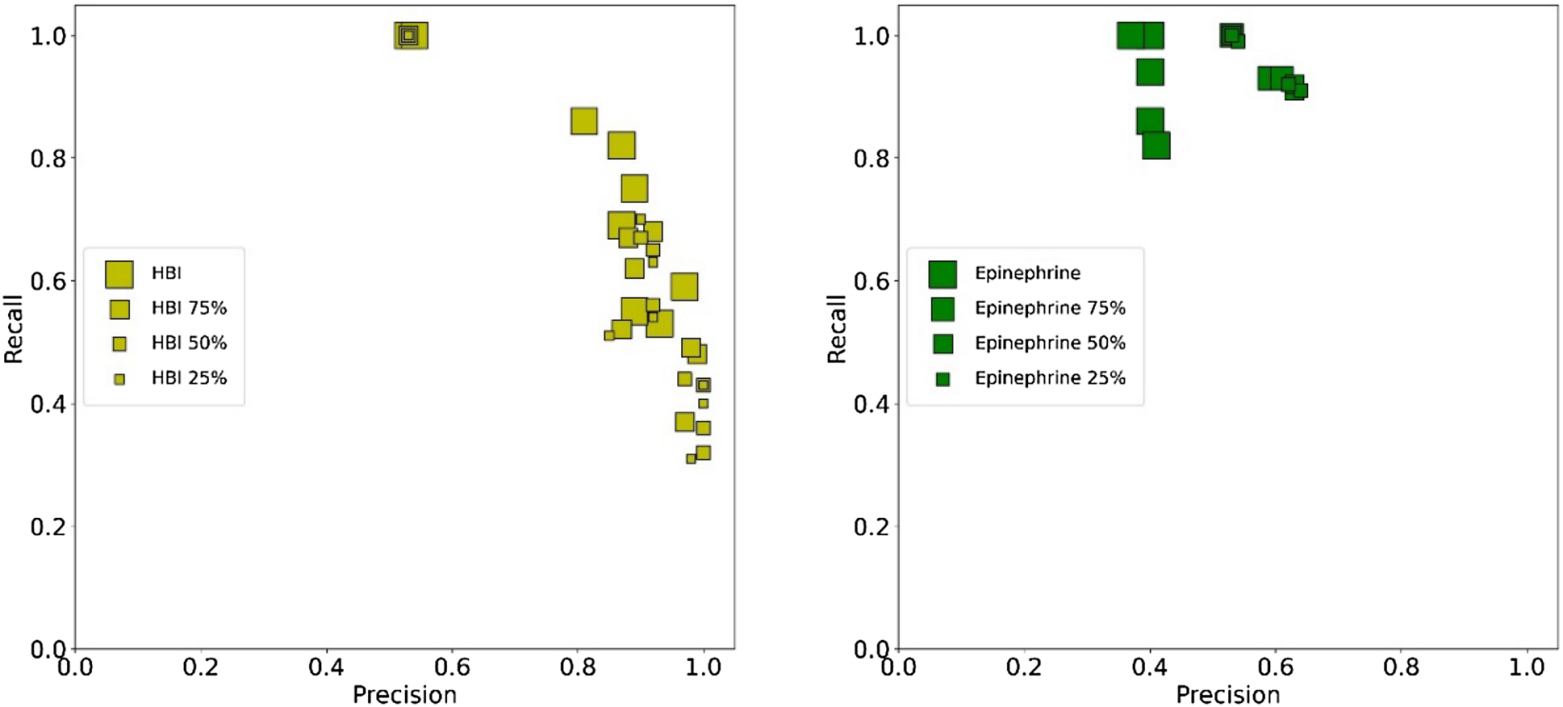
Average performance of the OCSVM models obtained for HBI, and epinephrine reference trajectories of variable length on the agonist/antagonist dataset from docking using the representative structures. Symbols size is proportional to the simulation length, ranging from 125 ns (25%) to 500 ns (100%)

The truncated simulation data was obtained by merging the selected frames in the three replicates. Applying the newly trained models on the agonist/antagonist dataset revealed that the number of selected molecules decreases as the MD simulation length is shortened, which is reflected by an increase in precision and a decrease in recall (Fig. 4 and Additional file 1: Tables S7, S8, S9, S10). The effect is much more marked if HBI is used as a reference, thus confirming that the epinephrine-ADRB2 trajectory does not highlight the interaction patterns that are crucial for the activation of the receptor.

#### Combination of multiple references

**Fig. 5:**
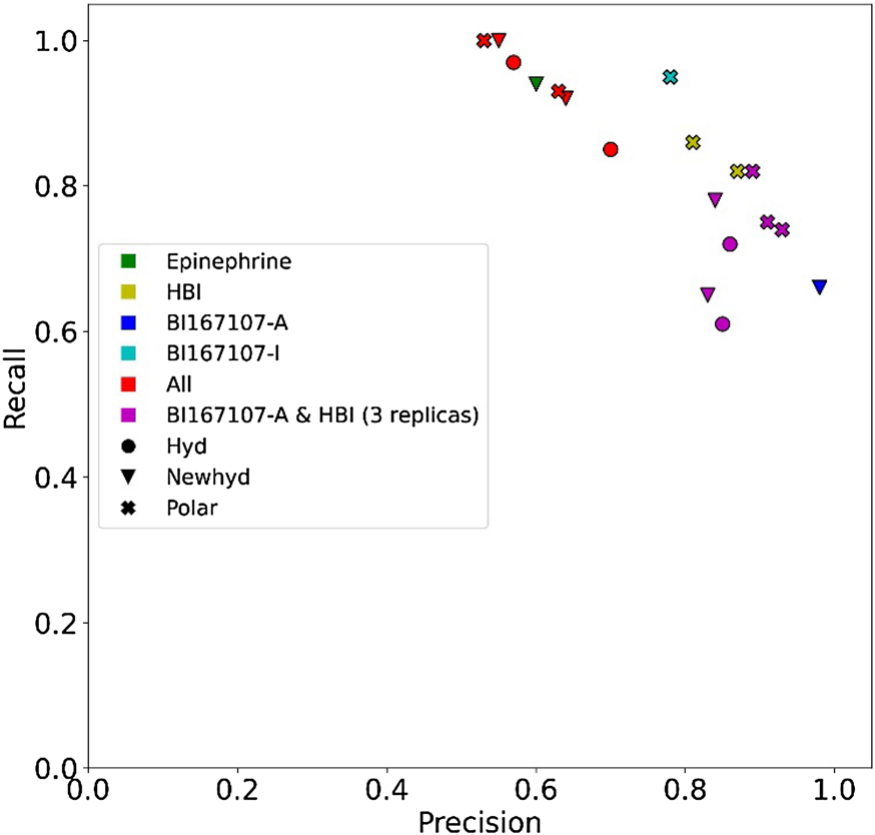
Average performance on the agonist/antagonist dataset of OCSVM models obtained from docking using the representative structures from all merged reference trajectories or HBI and BI167107-A. For the sake of comparison are also shown the best models obtained for epinephrine, HBI, BI167107-A, and BI167107-I, as given in Table 2

Although the proposed workflow was developed to use a single agonist as reference, the effect of merging multiple reference trajectories was investigated. We tested two combinations of datasets (Fig. 5 and Additional file 1: Tables S11, S12): All trajectories, BI167107-A and HBI. The models based on all the reference trajectories are characterized by a high recall and a relatively low precision. These poor performances are comparable to those obtained with the least relevant individual reference trajectory (that of epinephrine), suggesting that the determined agonist definition is too broad to be useful. The inclusion of BI167107-A data alongside the HBI trajectory increased the recall of the models including hydrophobic contacts. The overall effect of merging the two trajectories was however limited considering the performances of all models. This result further suggests that agonist-ADRB2 interactions in HBI and BI167107-A trajectories define a consistent binding mode.

### Comparison with known methods

The comparison between the binding modes of a reference ligand with the docking poses has already shown to be an effective method to improve the performances of virtual screening. To evaluate the performances of the proposed model with the state of the art we performed a rescoring of the docking poses of the agonist/antagonist and agonist/inactive datasets using IFP similarity, GRIM, and structure-based 3D pharmacophores. Here, given the docking poses generated using an ADRB2 structure extracted from a MD trajectory, the agonist binding mode observed in the selected frame was used as a reference for the three methods to rescore the corresponding set of docking poses.

GRIM and OCSVM models are based on the same description of interactions. GRIM scores docking poses using an empirical function evaluating the maximum common subgraph between the reference IG and the docking pose’s IG. For GRIM four different score thresholds have been proposed separating similar from dissimilar binding modes: 0.59, 0.65 [36], 0.70, 1.00 [47]. None of the four thresholds yielded satisfying results overall, with only a small increase in precision over selecting all ligands as agonist (eg average precision around 0.60 at threshold 0.65 for HBI and BI167107), or an extremely low recall (eg average recall of 0.10 at threshold 1.00 for HBI and BI167107-A) on both the dataset containing agonists and antagonists (Table 4 and Additional file 1: Tables S13–S16), and the dataset containing agonists and true inactive molecules (Additional file 1: Tables S17–S20). The difficulty in identifying a suitable GRIM score threshold to distinguish agonists is valid regardless of the set of docking poses considered (average results using a single representative structure, results using all representative structures, and results using the crystallographic structure).

**Table 4:** GRIM results on the agonists-antagonist dataset from docking data obtained using the representative structures or the crystallographic structure

Ligand	Threshold	Average		Crystallographic		Ensemble	
		Precision	Recall	Precision	Recall	Precision	Recall
Epinephrine	0.59	0.55	0.97	0.53	1.00	0.53	1.00
	0.65	0.70	0.62	0.63	1.00	0.56	1.00
	0.70	0.84	0.30	0.86	1.00	0.63	0.89
	1.00	–	0.00	–	0.00	–	0.00
HBI	0.59	0.53	1.00	0.53	1.00	0.53	1.00
	0.65	0.60	0.99	0.59	1.00	0.53	1.00
	0.70	0.67	0.98	0.68	1.00	0.53	1.00
	1.00	1.00	0.10	–	0.00	1.00	0.37
BI167107 active	0.59	0.53	1.00	0.53	1.00	0.53	1.00
	0.65	0.58	1.00	0.59	1.00	0.53	1.00
	0.70	0.64	0.99	0.68	1.00	0.58	1.00
	1.00	1.00	0.10	1.00	0.47	1.00	0.26
BI167107 inactive	0.59	0.53	1.00			0.53	1.00
	0.65	0.61	0.94			0.58	1.00
	0.70	0.69	0.84			0.70	1.00
	1.00	1.00	0.02			1.00	0.05

The standard definition of hydrophobic contacts is applied. Thresholds discarding all docking poses are indicated as –.

The different methods, GRIM, IFP, and pharmacophore search, were tested to determine the existence of a ligand independent threshold for classification. The optimal scoring value separating agonists and antagonists was determined for each ligand (Table 5 and Additional file 1: Table S21), as the threshold maximizing the F1 measure for the agonist class. All methods can be used to bias the screening towards agonists over antagonists, but no method specific score threshold can be identified from the results. The overall performances depend on the reference agonist rather than the method used for rescoring, but even for the same reference agonist it is difficult to determine a specific threshold since each receptor structure used for docking was characterized by its own optimal threshold. In summary, the three methods well performed in prioritizing agonists, however it is not possible to clearly define the threshold separating agonists from antagonists a priori.

**Table 5:** Optimal average scoring threshold for GRIM, IFP similarity, and 3D pharmacophore based on the agonist–antagonist dataset

Ligand	Model	Threshold	Precision	Recall
Epinephrine	GRIM	0.63	0.66	0.93
	GRIM–Newhyd	0.63	0.66	0.90
	IFP	0.38	0.64	0.93
	IFP–Polar	0.48	0.65	0.94
	FitValue	0.59	0.61	0.92
	Pharmtype	1.43	0.60	0.92
HBI	GRIM	0.76	0.82	0.91
	GRIM–Newhyd	0.75	0.81	0.91
	IFP	0.58	0.76	0.89
	IFP–Polar	0.57	0.71	0.92
	FitValue	2.00	0.87	0.80
	Pharmtype	4.00	0.79	0.80
BI167107 active	GRIM	0.82	0.91	0.92
	GRIM–Newhyd	0.77	0.89	0.92
	IFP	0.60	0.79	0.86
	IFP–Polar	0.58	0.80	0.89
	FitValue	2.16	0.93	0.84
	Pharmtype	4.00	0.76	0.88
BI167107 inactive	GRIM	0.73	0.75	0.95
	GRIM–Newhyd	0.71	0.74	0.97
	IFP	0.64	0.73	0.88
	IFP–Polar	0.67	0.76	0.84
	FitValue	1.52	0.74	0.93
	Pharmtype	4.50	0.78	0.78

## Conclusions

OCSVM models were developed from graphs encoding intermolecular interactions extracted from MD simulations of three reference complexes, between ADBR2 and epinephrine, HBI, and BI167107. Models based on HBI performed well in identifying agonists of the antagonist/agonist test set (best model average precision: 0.87), and filtering out inactive molecules of the agonist/inactive test set (best model average precision: 1.00). Failure in identifying agonists from the agonist/inactive test set was related to the substitution of hydrogen bond donor groups with chlorine and fluorine atoms. Similar results were obtained for BI167107-based models if they were trained from the portion of the MD trajectory that describes the active state of ADRB2. Performance deteriorated for models trained with the portion of the MD trajectory that describes the inactive state of ADRB2. Epinephrine-based models displayed low precision, tending to predict as agonists all molecules from both datasets. This poor performance was explained by an inappropriate sampling of the agonist binding mode, with epinephrine showing variable interactions patterns during simulation, probably including unbinding initiation. The importance of agonist binding mode sampling was also revealed by the effect of the MD simulation length on the models’ performances. The OCSVM models were compared to established approaches for the post-processing of virtual screening results that search for the reference binding mode in docking poses: matching of interactions graphs using GRIM, comparing interaction fingerprints using IFP, and searching for 3D pharmacophores. All methods prioritized agonist ligands over inactive molecules and antagonists, yet only OCSVM models have a built-in threshold separating the two classes. With the three other methods ranking molecules in the dataset, a test set is required to properly define a scoring threshold, thus restraining their application to already well-studied GPCRs.

**Fig. 6:**
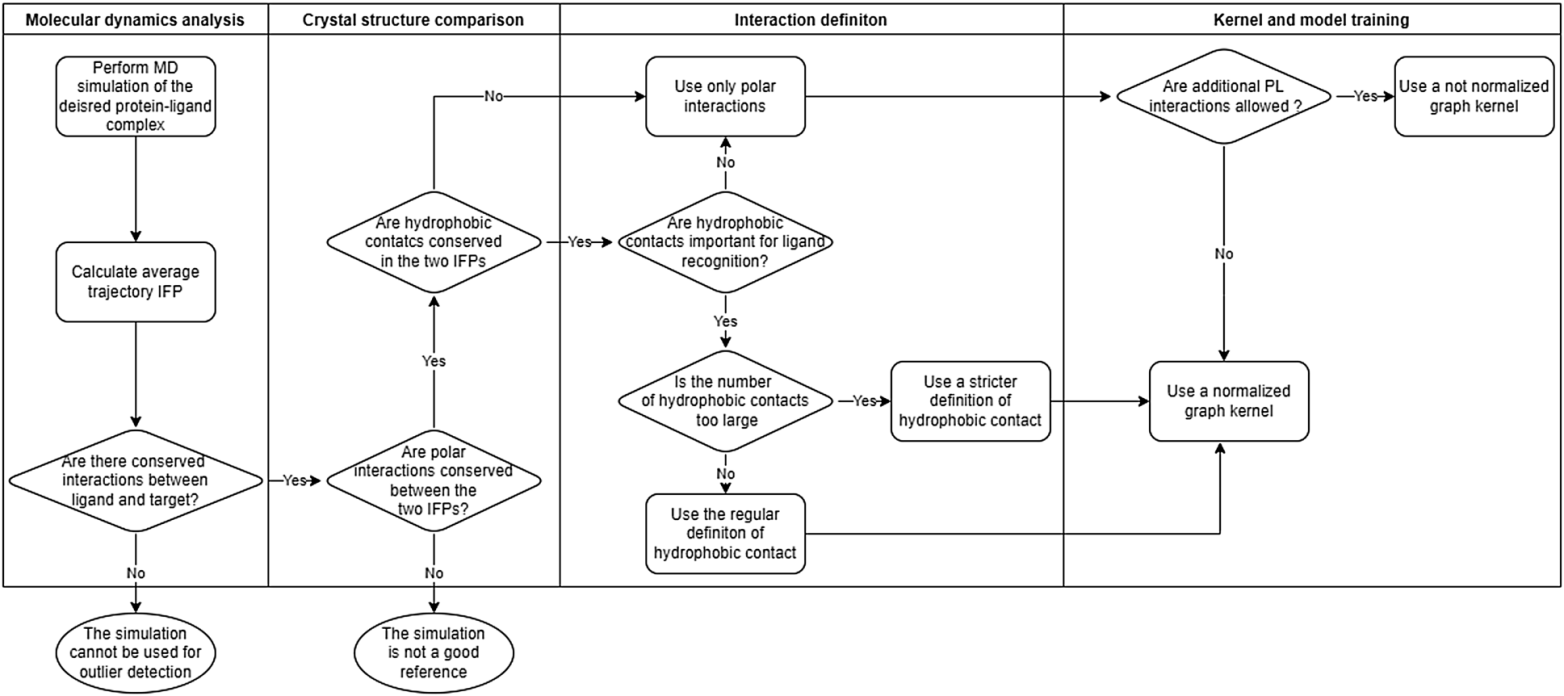
Flowchart for model development

The proposed method can be applied to any GPCR provided that a three-dimensional structure is available between this GPCR and an agonist, and the corresponding MD simulation correctly samples the binding mode. Basic guidelines on how to build an appropriate model are given on Fig. 6 and can be applied to other targets.

## Materials and methods

### Molecular dynamics simulations

The MD trajectories of ADRB2 bound to epinephrine and HBI were downloaded from GPCRmd [32]. The ADRB2-Epinephrine (ID 117) simulation is based on the PDBID:4LDO [34] crystallographic structure. The protein is embedded in a lipid bilayer formed by POPC, the system is solvated by an aqueous solution of Na + (158 mM) and Cl − (184 mM).

The ADRB2-HBI (ID 115) simulation is based on the PDBID:4LDL [34]crystallographic structure. The protein is embedded in a lipid bilayer formed by POPC, the system is solvated by an aqueous solution of Na + (156 mM) and Cl − (182 mM).

Both simulations were performed using ACEMD with GPUGRID, the used forcefield is CHARMM36m. Three replicas of 0.5 μs were performed for each system with a timestep of 4.0 fs and a gap between the saved frames of 0.2 ns. For each system a dataset of 7500 protein–ligand structures is available.

The ADRB2-BI167107 simulation was performed by Dror et al.[33] using as starting structure the PDBID:3P0G [48] crystallographic structure. The protein is embedded in a lipid bilayer formed by POPC; the system is solvated by an aqueous solution of NaCl (0.15 M). The simulation was performed on Anton, the used forcefield is CHARMM27.

The entire trajectory (trajectory number 11 in the original publication) represents 10 μs of simulation, with frames saved every 180 ps. The part of the trajectory representing the active state receptor bound to the ligand corresponds to the first 350 ns of simulation. The part of trajectory used to represent the inactive state receptor begins after 3.60 μs of simulation and ends at 4.95 μs. The active state receptor dataset corresponds to 1944 frames, while the inactive state receptor dataset corresponds to 7500 frames.

### Crystallographic structure preparation

The three reference crystallographic structures (PDBID: 3P0G, 4LDO, 4LDL) were downloaded from the PDB online repository. The structures were protonated and converted to mol2 files using MOE 2020. Protonation state and tautomer state of the residues were selected to match the one observed in the corresponding MD simulations.

### Binding mode analysis

The agonist-ADRB2 binding modes depicted on the top of Fig. 1 are a representation of the interactions detected using MOE 2020.

The binding of each ligand during the simulation, as shown on Fig. 1, was analysed by computing IFP using IChem. The average IFP over each trajectory was calculated. Interactions observed in at least 10% of the frames of a single simulation, or observed in the crystallographic structure, are shown in the IFP graphical representation.

### Interaction detection

Protein–ligand interactions were computed using IChem v5.2.9 [36]. IChem differentiates interactions according to the following seven types: Hydrogen bonds with the protein acting as acceptor, hydrogen bond with the protein acting as donor, ionic interactions, separated depending on the charge of the protein and the ligand, aromatic stacking, grouping together edge-to-face and face-to-face interactions, hydrophobic contacts, and metal chelation. An interaction is detected between two atoms depending on their types and on topological constrains. Hydrogen bonds and aromatic stacking have both a distance and an angle constrain, while the remaining interaction depend only on the distance [36]. IChem presents two definitions of hydrophobic contacts, Hyd and Newhyd. The Hyd definition considers hydrophobic contacts between all atoms with the correct atom types, while the Newhyd definition considers only atoms with the correct atom types and in a hydrophobic environment.

IChem requires as input mol2 files with precise characteristics. The MD trajectory was converted in a series of pairs of mol2 files containing the protein and the ligand, removing lipids, water molecules, and ions. Such conversion was performed using a Python script based on pytraj [49, 50] for mol2 file generation. The generated files were corrected by a Python script to be compatible with IChem: CHARMM atom types were converted to SYBYL atom types, and the bond order between atoms was determined based on the atom types. Interaction detection was performed using the default definition of hydrophobic contacts and a stricter definition (-Newhyd). The definition of aromatic interactions was changed increasing the threshold distance to 5.0Å (−D_Ar 5.0).

### Model building

#### Graph kernel

**Fig. 7:**
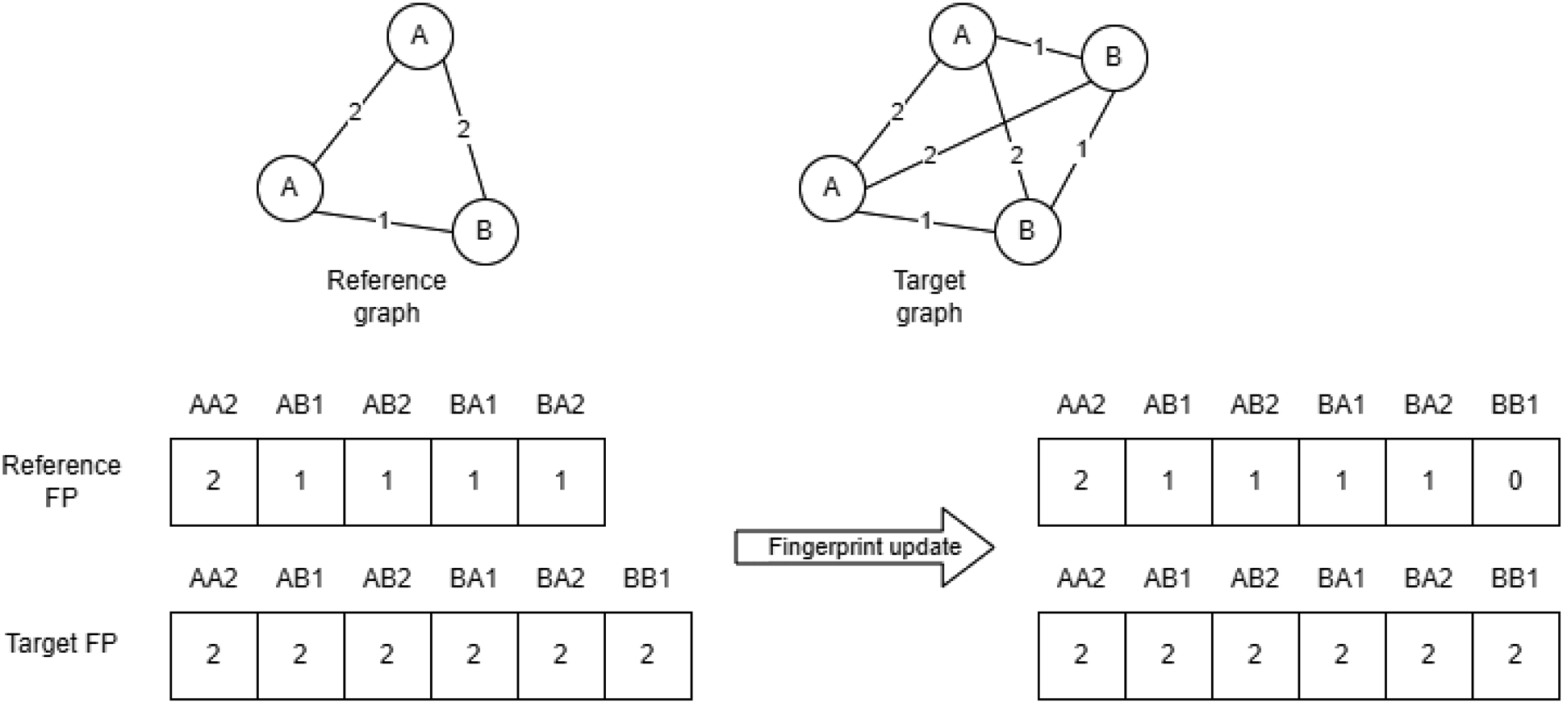
Fingerprint generation and update for shortest path kernel similarity calculation

Graph similarity was evaluated using the shortest path kernel implemented in GraKel [51]. The shortest path kernel generates from the input graph the corresponding shortest path graph. The shortest path graph is a fully connected graph, with edges labelled according to the length of the shortest path between two nodes in the original graph. The shortest path graph is encoded as a fingerprint. Each element of the fingerprint corresponds to a pair of node labels and the label of the edge connecting them. The fingerprint contains the frequency of each path in the graph. There is no predefined fingerprint to describe the graphs, the fingerprint is learned from the training dataset to contain the observed paths. The fingerprint is temporarily updated during kernel application on new graphs to include previously unseen paths. The update in the fingerprint is relevant only for normalized similarity score calculations (Fig. 7).

#### Model training

The Scikit-learn [52] implementation of the OCSVM algorithm was used for the classifier. The model was trained using two different heuristics: QMS2 and a MAD based method. For both methods the average KNN similarity was calculated, with K corresponding to 3% of the number of instances in the dataset.

For QMS2 the KNN-similarity values were sorted in ascending order. A function was interpolated linking the rank of each point to its similarity score. Outliers are defined as points before the first major knee. For such points an increase in rank is associated with a rapid increase of KNN similarity. The knee was determined using the kneedle algorithm [53], selecting the first concave point. Kneedle sensibility parameter was manually selected to identify the true global knee of the function. Points below the selected knee were discarded and ν = 0.01.

For training based on MAD, the median and MAD values were calculated using SciPy [54]. The fraction of outliers in the training set was determined as the fraction of points with a distance from the median greater than 3 times MAD.

A Python script, available at https://github.com/LIT-CCM-lab/OCSVM-ADRB2, was developed for the training of the kernel and the determination of OCSVM parameters using both methods.

### Evaluation metrics

The performances of the presented classification methods are evaluated according to their precision, recall, and F1 measure[39, 55].

Precision and recall are defined as:

$$Precision= \frac{TP}{TP+FP}$$

$$Recall= \frac{TP}{TP+FN}$$

With TP (true positive) indicating agonist ligands correctly classified as agonist, FP (false positives) indicating antagonist or inactive molecules classified as agonist, and FN indicating agonist molecules not classified as agonist.

The classifier was trained to identify instances from a single class, the F1 score calculated on the positive class (agonist) was selected as a metric to evaluate the overall performance of the model.

The F1 measure is defined as:

$$F1=2\frac{Precision*Recall}{Precision+Recall}$$

### Selection of representative conformations

#### Binding site definition

Two definitions of the receptor binding site have been used in this work, a system specific definition and a shared definition. The system specific binding site is obtained by selecting all non-hydrogen atoms at less than 4.5Å from the non-hydrogen atoms of the ligand for at least 10% of the trajectory frames. The shared definition includes all residues containing at least one atom present in one of the four different system specific binding site definitions.

#### Trajectory clustering

Similarity between receptor conformations was evaluated using the RMSD of interacting atoms.

$$RMSD= \sqrt{\frac{{\sum }_{i}^{N}{({X}_{i}-{Y}_{i})}^{2}}{N}}$$

With *N* indicating the number of selected interacting atoms, *X*_*i*_ and *Y*_*i*_ the coordinates of each atom *i* in the two compared structures. The pairwise RMSD between structures in the same trajectory was computed, on atoms in the system specific binding site definition, using pytraj. The pairwise distances were represented by a histogram to determine the presence of a clusterable data structure[56].

Only the ADRB2-HBI trajectory presents multiple clearly distinct conformation of the binding site based on the RMSD. Agglomerative hierarchical clustering with average linkage was performed on the HBI-ADRB2 dataset using the scikit-learn implementation of the algorithm. The optimal number of clusters was evaluated considering different geometrical criteria, as already proposed by De Paris et al. [57]: Silhouette score, Dunn index, and Davies-Bouldin index. The optimal number of clusters was estimated at two, with a major cluster (6881 elements) and a minor cluster (619 elements).

The remaining trajectories and the major cluster of the HBI-ADRB2 complex were clustered using the K-medoids algorithm [58], implemented in scikit-learn-extra. The optimal number of clusters was estimated according to geometrical criteria: Silhouette score, Dunn index, and Davies-Bouldin index. From HBI-main-cluster 9 clusters were obtained, 8 for epinephrine, 2 for BI167107-I, and 4 for BI167107-A. For each cluster the medoid, the member of the cluster with the lowest distance from all other members of the same cluster, was selected as the representative structures.

#### Comparison to the crystallographic structure

The RMSD between the starting crystal structure and the trajectory was evaluated using the shared definition of binding site. The RMSD was calculated using pytraj. The shared definition of binding site was used to have comparable values between the trajectories.

#### Pairwise comparison of the trajectories

The pairwise RMSD of the representative structures and of the trajectories was calculated using the common definition of the binding site. For the comparison between the trajectories one every five saved frames was kept for analysis.

### Agonist–antagonist validation set preparation

#### Initial ligand selection

The initial set of molecules was selected from the ADRB2 (CHEMBL210) target page on ChEMBL [59], in the section “Drugs and Clinical Candidates”, 33 agonists, 2 partial agonists and 13 antagonists were retrieved. An additional literature search was performed to increase the number of antagonists in the dataset, with the inclusion of 7 new molecules, all explicitly described as ADRB2 antagonist. Bupranolol, tertatolol, IPS339, spirendolol, ICI118551 were selected from an article comparing the electronic structure of β-receptor agonist and antagonist [60]. Compound CHEMBL355038 (15a) [61] and CHEMBL3228930 (4)[62] were retrieved from articles describing their synthesis. Ligands with “Mechanism of action” antagonist from GPCRdb [63] “Drugs target and Indications” targeting ADRB2 were selected. Only new entries with literature reference were considered. Three ligands (Alprenolol, Carteolol, Carvedilol) were added to the dataset. From the IUPHAR/BPS Guide to Pharmacology page on ADRB2 [64] four new antagonist ligands were added to the dataset (Butoxamine, CGP-12177, SR59230A, NIP). The initial dataset contains 29 agonists, 27 antagonists, and 2 partial agonists.

#### Dataset cleaning

Duplicates corresponding to specific enantiomers of already present racemic ligands were discarded.

Three ligands with unknown structure were removed (LAS190792, AZD3199, GSK159802). The two ligands described as partial agonist (pindolol, celiprolol) were also discarded from the dataset. The number of heavy atoms of each ligand was calculated using RDKit[65]. Molecules with a significantly larger number of heavy atoms (Z-score > 1.5) were discarded to have a dataset of molecules with homogeneous size.

Morgan’s structural fingerprints were calculated for the remaining molecules with a radius of 2 and a fingerprint length of 2048 bits using RDKit. The pairwise Tanimoto distance (1 – Tanimoto similarity) was computed and used to perform agglomerative hierarchical clustering. A distance threshold of 0.5 was set for the clustering. From each cluster the molecule with the highest affinity for the target was selected. The highest affinity was determined by comparing results from assays performed in similar conditions. In case no comparable assay results were found, the best characterized molecule was selected.

The scaffold distribution between agonist and antagonists was evaluated by retrieving the Bemis-Murcko scaffolds with RDKit.

### Ligand preparation

The ligands, represented as SMILES, were ionized using the FILTER program from OpenEye (Filter 2.5.1.4 OpenEye Scientific Software, Santa Fe, NM, USA). The ligands’ 3D structures were generated using Corina 3.40 (Molecular Networks GmbH, Nürnberg, Germany)[66]. The *rc* option was used to generate multiple ring conformation. For ligands presenting stereocenters all stereoisomers were generated.

### Docking

Rigid protein ligand docking was performed using PLANTS v1.2, using the CHEMPLP scoring function and search speed 1 (highest accuracy) [67]. The cavity centre of each protein was identified as the centre of mass of the interacting atoms in the system specific definition of the binding site. The cavity radius was set to 12 Å for all structures. For each ligand stereoisomer 10 poses were saved.

### Docking post processing

#### IFP

Interaction fingerprints were calculated using IChem. Comparison between fingerprints was performed using a Python script. Interaction similarity was defined as the Tanimoto similarity between the two binary IFPs.

#### GRIM

Protein–ligand interactions were detected using IChem, with both available definitions of hydrophobic contacts. For each docking pose the generated IPAs were saved in mol2 files. The definition of aromatic interactions was changed increasing the threshold distance to 5.0 Å (-D_Ar 5.0). The GRIM score between the docking IPAs and the reference IPAs was computed using IChem.

#### 3D pharmacophore

Interaction 3D pharmacophores were generated automatically using DiscoveryStudio (Dassault Systemes BIOVIA), the settings for generation were changed to include all the detected interactions between ligand and protein as pharmacophoric features.

The software *citest* was used for evaluation of the docking pose by comparing the alignment to the pharmcophoric query without performing fitting. Two different scores were used to evaluate the poses: FitScore, which is a sum of the fitting quality to each feature, and Pharmtype, which measures the number of features of the query matched by the pose.

#### OCSVM evaluation

The IPA files generated from the MD trajectory were used to train the shortest path graph kernel and the OCSVM classification model. The docking poses IPAs were converted to IGs. The node labels were extracted from the residue name of the IPAs. The adjacency matrix was calculated as the pairwise Euclidean distances between the IPAs, approximated to the nearest Å. A distance of 0.1 was set for overlapping IPAs. Polar IGs were generated from regular interaction files by removing hydrophobic IPAs. The similarity to the graphs in the training set was calculated using the trained shortest path graph kernel. The obtained Gram matrix is used by the OCSVM model to score the IGs. Negative scores indicate an outlier, while positive scores indicate an inlier. Inliers are considered as active molecules with the same pharmacological effect as the reference.

## Supplementary Information

**Additional file 1: Figure S1. **Average and crystallographic interaction fingerprints for the three reference agonists. **Figure S2.** RMSD of the binding site residues during the simulation of the BI167107-ADRB2 complex. **Figure S3.** Pairwise RMSD of the four reference trajectories. **Figure S4. **Pairwise RMSD of the representative structures used for docking. **Figure S5.** RMSD of the binding site residues during the simulation of the epinephrine-ADRB2 complex. **Figure S6.** RMSD of the binding site residues during the simulation of the HBI-ADRB2 complex. **Table S1. **Annotated list of molecules from the agonist/antagonist dataset. **Table S2. **Annotated list of molecules from the agonist/inactive dataset. **Table S3. **Average performances of the OCSVM models on the agonist/antagonist dataset, from docking data obtained using the representative structures. **Table S4. **Performances of the OCSVM models on the agonist/antagonist dataset, from docking data obtained using crystallographic structures. **Table S5.** Average performances of the OCSVM models on the agonist/inactive dataset, from docking data obtained using the representative structures. **Table S6.** Performances of the OCSVM models on the agonist/inactive dataset, from docking data obtained using crystallographic structures. **Table S7. **Average performances of shorter trajectories of epinephrine-based OCSVM models on the agonist-antagonist dataset, from docking data obtained using the representative structures. **Table S8.** Performances of shorter trajectories of epinephrine-based OCSVM models on the agonist-antagonist dataset, from docking data obtained using crystallographic structures. **Table S9. **Average performances of shorter trajectories of HBI-based OCSVM models on the agonist-antagonist dataset, from docking data obtained using the representative structures. **Table S10. **Performances of shorter trajectories of HBI-based OCSVM models on the agonist-antagonist dataset, from docking data obtained using crystallographic structures. **Table S11.** Average performances of the combined dataset OCSVM models on the agonist-antagonist dataset, from docking data obtained using the representative structures. **Table S12. **Performances of the combined dataset OCSVM models on the agonist-antagonist dataset, from docking data obtained using crystallographic structures. **Table S13. **Average performance of GRIM at different thresholds on the agonist-antagonist dataset, from docking data obtained using the representative structures. Default definition of hydrophobic contacts. **Table S14. **Performance of GRIM at different thresholds on the agonist-antagonist dataset, from docking data obtained using crystallographic structures. Default definition of hydrophobic contacts. **Table S15. **Average performance of GRIM at different thresholds on the agonist-antagonist dataset, from docking data obtained using the representative structures. Newhyd definition of hydrophobic contacts. **Table S16.** Performance of GRIM at different thresholds on the agonist-antagonist dataset, from docking data obtained using crystallographic structures. Newhyd definition of hydrophobic contacts. **Table S17.** Average performance of GRIM at different thresholds on the agonist-inactive dataset, from docking data obtained using the representative structures. Default definition of hydrophobic contacts. **Table S18.** Performance of GRIM at different thresholds on the agonist-inactive dataset, from docking data obtained using crystallographic structures. Default definition of hydrophobic contacts. **Table S19.** Average performance of GRIM at different thresholds on the agonist-inactive dataset, from docking data obtained using the representative structures. Newhyd definition of hydrophobic contacts. **Table S20.** Performance of GRIM at different thresholds on the agonist-inactive dataset, from docking data obtained using crystallographic structures. Newhyd definition of hydrophobic contacts. **Table S21.** Average optimal threshold for agonist/antagonist classification for GRIM, IFP, and 3D pharmacophore. **Table S22.** Interaction graphs statistics from the four reference trajectories. **Table S23.** Interaction graphs statistics form the docking poses of the agonist-antagonist dataset and agonist-inactive datasets.

## Data Availability

The training data (IPA, IG) and the test datasets (annotated structures and docking poses) are accessible via an online repository (https://seafile.unistra.fr/d/548138136ff14e3889c1). The code and protocol required to develop the model starting from the MD trajectory is available at: https://github.com/LIT-CCM-lab/OCSVM-ADRB2.

## Abbreviations

ADRB2: β2 Adrenergic receptor
COPD: Chronic obstructive pulmonary disease
MD: Molecular dynamics
OCSVM: One class support vector machine
KNN: K-nearest neighbors
HBI: Hydroxybenzyl isoproterenol
BI167107-A: BI167107 bound to the active state of ADRB2
BI167107-I: BI167107 bound to the inactive state of ADRB2
Hyd: Regular definition of hydrophobic contacts in IChem
Newhyd: Stricter definition of hydrophobic contacts in IChem
IG: Interaction graph
NN: Not normalized
SV: Support vector
QMS2: Quick model selection 2
MAD: Median absolute deviation
IFP: Interaction fingerprints
GRIM: Graph matching of interaction patterns
RMSD: Root mean square deviation
