# Supplementary material for: One class classification for the detection of β2 adrenergic receptor agonists using single-ligand dynamic interaction data
Source: J Cheminform. 2022 Oct 29;14:74. doi: 10.1186/s13321-022-00654-z (PMC9617447; doi:10.1186/s13321-022-00654-z)
Supplement: Supplementary file 1 — Additional file 1: Figure S1. Average and crystallographic interaction fingerprints for the three reference agonists. Figure S2. RMSD of the binding site residues during the simulation of the BI167107-ADRB2 complex. Figure S3. Pairwise RMSD of the four reference trajectories. Figure S4. Pairwise RMSD of the representative structures used for docking. Figure S5. RMSD of the binding site residues during the simulation of the epinephrine-ADRB2 complex. Figure S6. RMSD of the binding site residues during the simulation of the HBI-ADRB2 complex. Table S1. Annotated list of molecules from the agonist/antagonist dataset. Table S2. Annotated list of molecules from the agonist/inactive dataset. Table S3. Average performances of the OCSVM models on the agonist/antagonist dataset, from docking data obtained using the representative structures. Table S4. Performances of the OCSVM models on the agonist/antagonist dataset, from docking data obtained using crystallographic structures. Table S5. Average performances of the OCSVM models on the agonist/inactive dataset, from docking data obtained using the representative structures. Table S6. Performances of the OCSVM models on the agonist/inactive dataset, from docking data obtained using crystallographic structures. Table S7. Average performances of shorter trajectories of epinephrine-based OCSVM models on the agonist-antagonist dataset, from docking data obtained using the representative structures. Table S8. Performances of shorter trajectories of epinephrine-based OCSVM models on the agonist-antagonist dataset, from docking data obtained using crystallographic structures. Table S9. Average performances of shorter trajectories of HBI-based OCSVM models on the agonist-antagonist dataset, from docking data obtained using the representative structures. Table S10. Performances of shorter trajectories of HBI-based OCSVM models on the agonist-antagonist dataset, from docking data obtained using crystallographic structures. Table S11. A [file 13321_2022_654_MOESM1_ESM.docx]

SUPPLEMENTARY INFORMATION

One class classification for the detection of β2 adrenergic receptor agonist using single-ligand dynamic interaction data

*Luca Chiesa^1^, Esther Kellenberger^1*^*

1 Laboratoire d’innovation thérapeutique, UMR7200 CNRS Université de Strasbourg, Faculté de Pharmacie, 67400 Illkirch-Graffenstaden, France.

SUMMARY

Figure 1: Average and crystallographic interaction fingerprints for the three reference agonists.

Figure 2: RMSD of the binding site residues during the simulation of the BI167107-ADRB2 complex.

Figure 3: Pairwise RMSD of the four reference trajectories

Figure 4: Pairwise RMSD of the representative structures used for docking

Figure 5: RMSD of the binding site residues during the simulation of the epinephrine-ADRB2 complex.

Figure 6: RMSD of the binding site residues during the simulation of the HBI-ADRB2 complex.

Table 1: Annotated list of molecules from the agonist/antagonist dataset.

Table 2: Annotated list of molecules from the agonist/inactive dataset.

Table 3: Average performances of the OCSVM models on the agonist/antagonist dataset, from docking data obtained using the representative structures.

Table 4: Performances of the OCSVM models on the agonist/antagonist dataset, from docking data obtained using crystallographic structures.

Table 5: Average performances of the OCSVM models on the agonist/inactive dataset, from docking data obtained using the representative structures.

Table 6: Performances of the OCSVM models on the agonist/inactive dataset, from docking data obtained using crystallographic structures.

Table 7: Average performances of shorter trajectories of epinephrine-based OCSVM models on the agonist-antagonist dataset, from docking data obtained using the representative structures.

Table 8: Performances of shorter trajectories of epinephrine-based OCSVM models on the agonist-antagonist dataset, from docking data obtained using crystallographic structures.

Table 9: Average performances of shorter trajectories of HBI-based OCSVM models on the agonist-antagonist dataset, from docking data obtained using the representative structures.

Table 10: Performances of shorter trajectories of HBI-based OCSVM models on the agonist-antagonist dataset, from docking data obtained using crystallographic structures.

Table 11: Average performances of the combined dataset OCSVM models on the agonist-antagonist dataset, from docking data obtained using the representative structures.

Table 12: Performances of the combined dataset OCSVM models on the agonist-antagonist dataset, from docking data obtained using crystallographic structures.

Table 13: Average performance of GRIM at different thresholds on the agonist-antagonist dataset, from docking data obtained using the representative structures. Default definition of hydrophobic contacts.

Table 14: Performance of GRIM at different thresholds on the agonist-antagonist dataset, from docking data obtained using crystallographic structures. Default definition of hydrophobic contacts.

Table 15: Average performance of GRIM at different thresholds on the agonist-antagonist dataset, from docking data obtained using the representative structures. Newhyd definition of hydrophobic contacts.

Table 16: Performance of GRIM at different thresholds on the agonist-antagonist dataset, from docking data obtained using crystallographic structures. Newhyd definition of hydrophobic contacts.

Table 17: Average performance of GRIM at different thresholds on the agonist-inactive dataset, from docking data obtained using the representative structures. Default definition of hydrophobic contacts.

Table 18: Performance of GRIM at different thresholds on the agonist-inactive dataset, from docking data obtained using crystallographic structures. Default definition of hydrophobic contacts.

Table 19: Average performance of GRIM at different thresholds on the agonist-inactive dataset, from docking data obtained using the representative structures. Newhyd definition of hydrophobic contacts.

Table 20: Performance of GRIM at different thresholds on the agonist-inactive dataset, from docking data obtained using crystallographic structures. Newhyd definition of hydrophobic contacts.

Table 21: Average optimal threshold for agonist/antagonist classification for GRIM, IFP, and 3D pharmacophore.

Table 22: Interaction graphs statistics from the four reference trajectories

Table 23: Interaction graphs statistics form the docking poses of the agonist-antagonist dataset and agonist-inactive datasets.


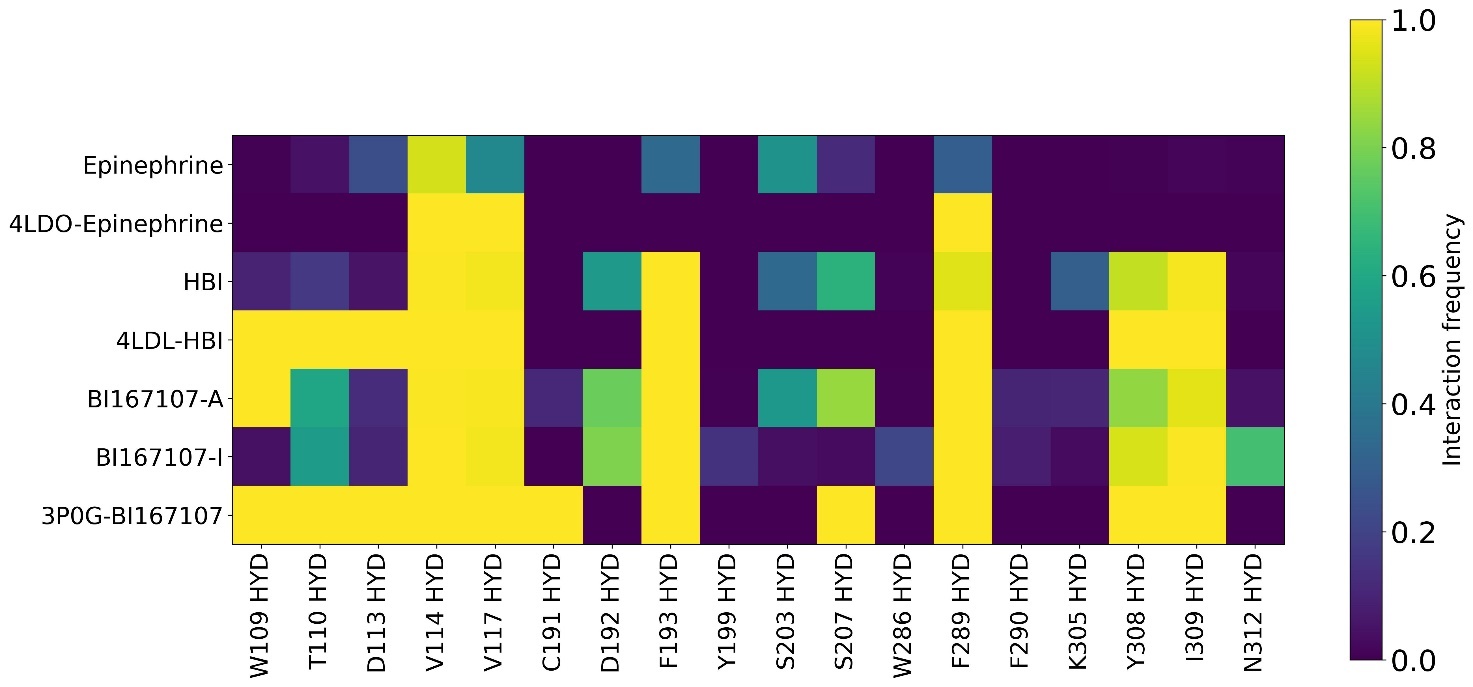


***SI Figure 1*** *Average number of hydrophobic contacts formed by the agonists with ADRB2 during the simulation, as compared to the hydrophobic contacts observed in the crystallographic structures.*


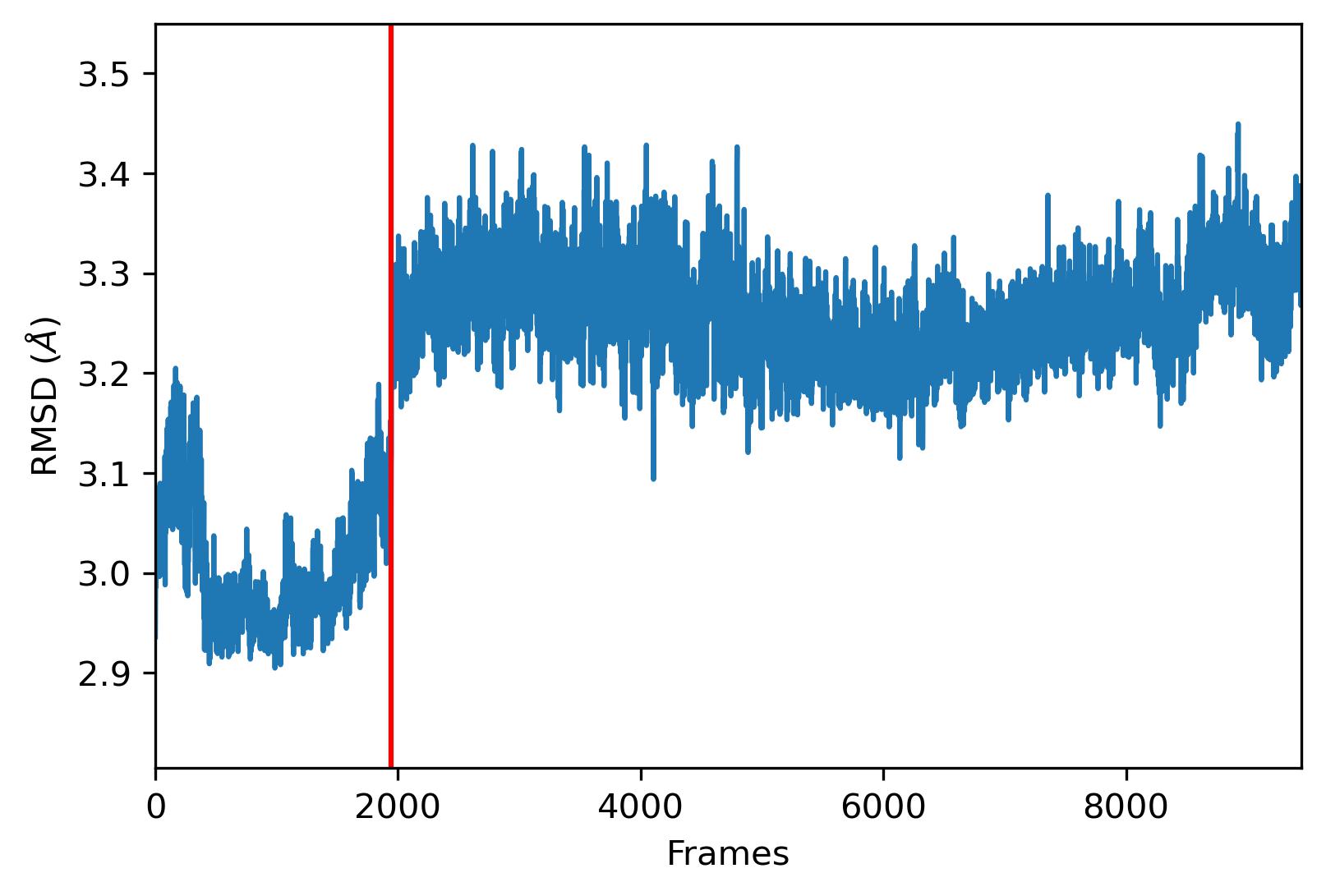


***SI Figure 2*** *RMSD of the atoms in the binding site from the crystallographic structure of the BI167107-ADRB2 trajectory. The red line separates the frames describing the active state of the receptor from the frames containing the inactive state receptor.*


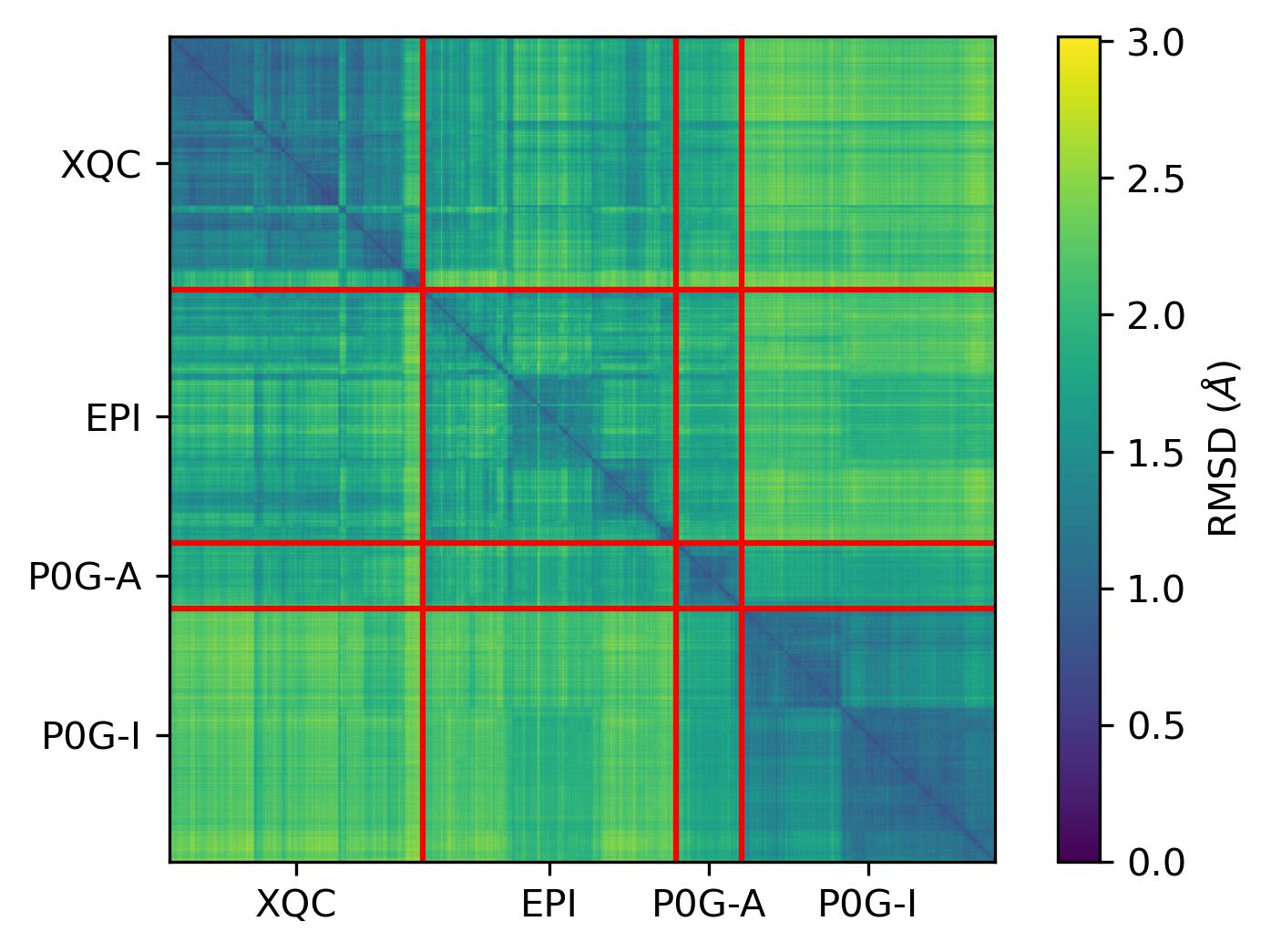


***SI Figure 3*** *Pairwise RMSD of the atoms in the binding site between the four simulations. The red lines separate the frames of the four simulations.*


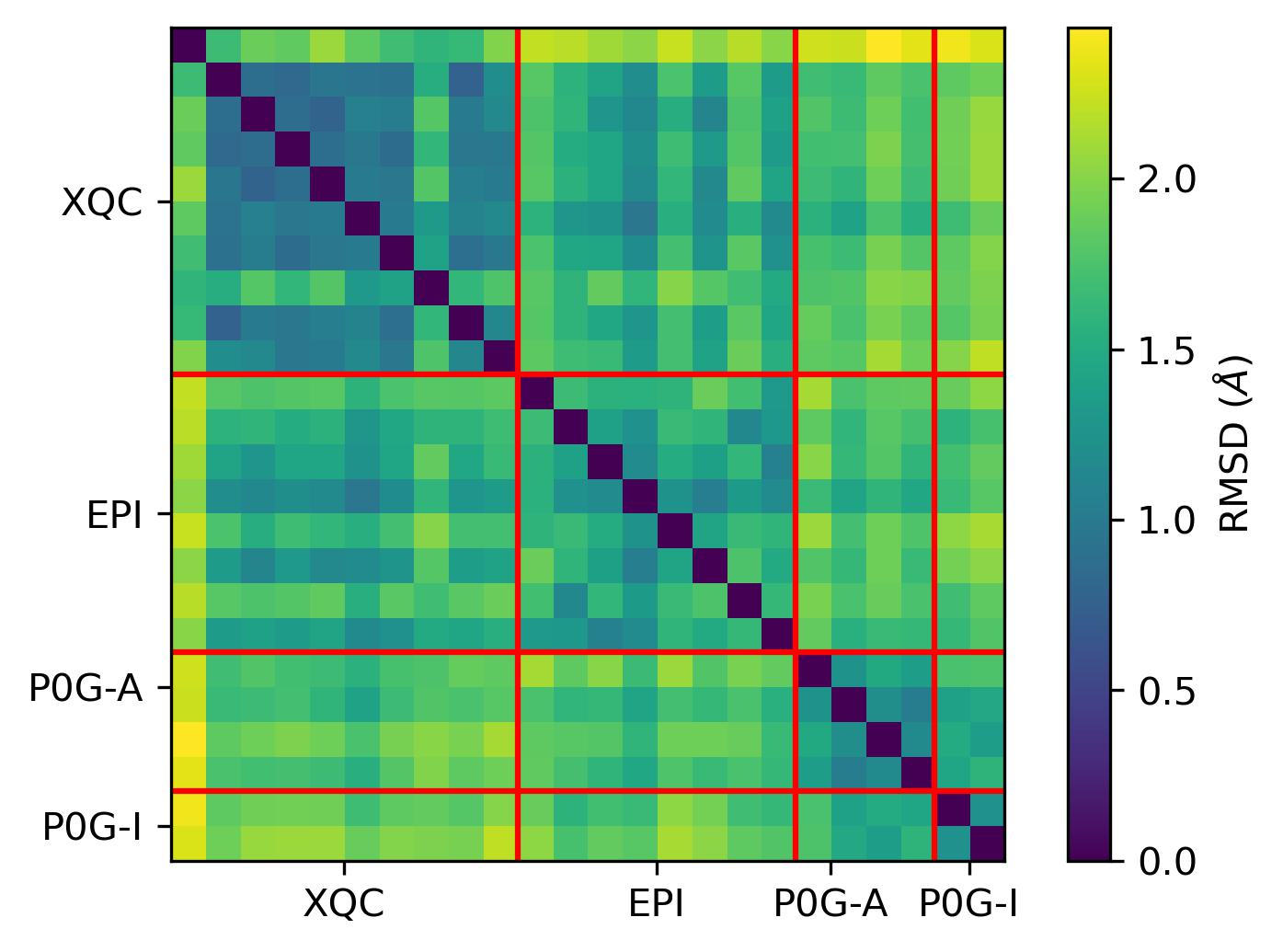


***SI Figure 4*** *Pairwise RMSD of the atoms in the binding site between the representative structures extracted from the four simulations. The red lines separate the frames of the four simulations.*


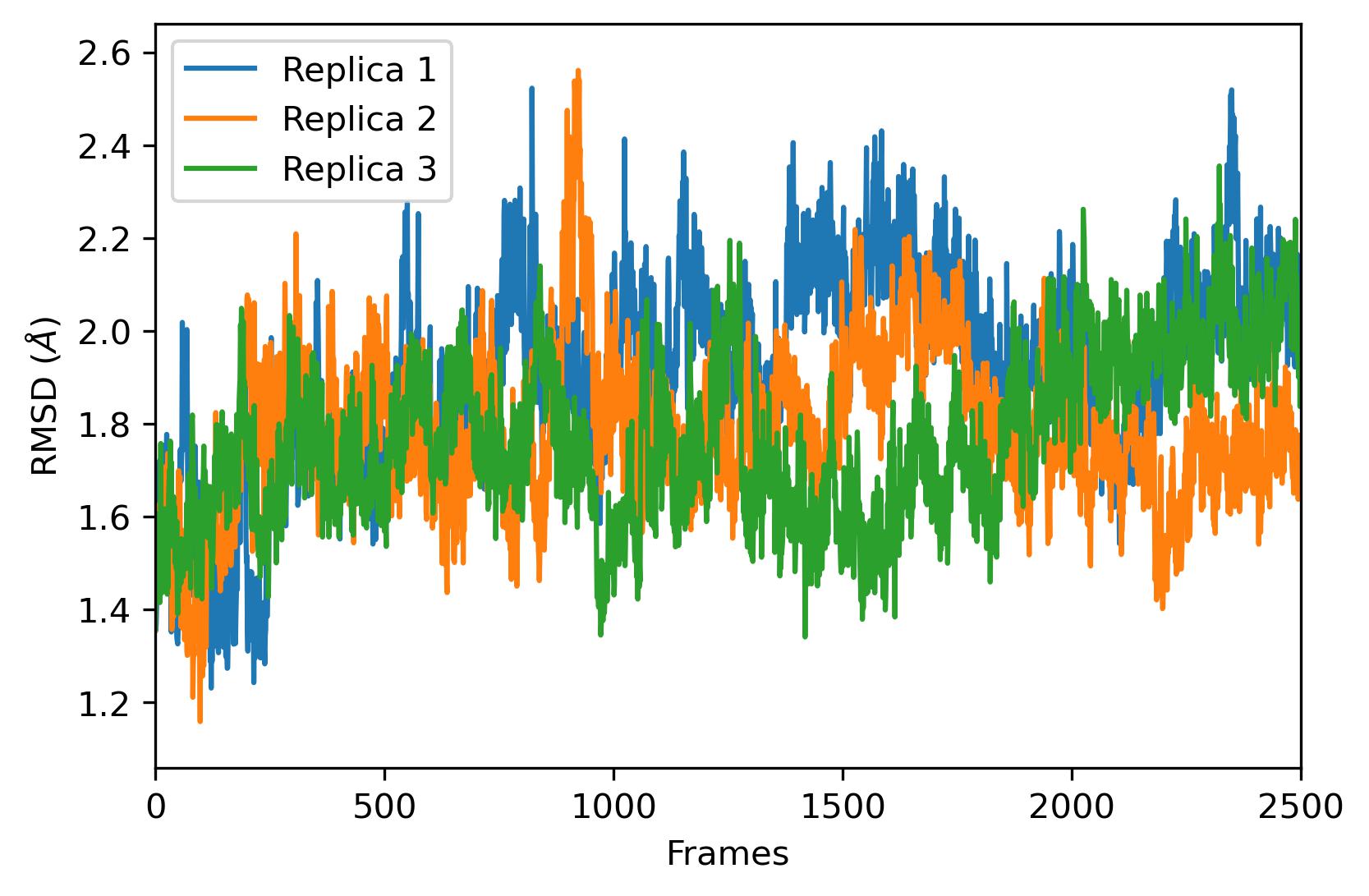


***SI Figure 5*** *RMSD of the atoms in the binding site from the crystallographic structure of the three epinephrine-ADRB2 trajectories.*


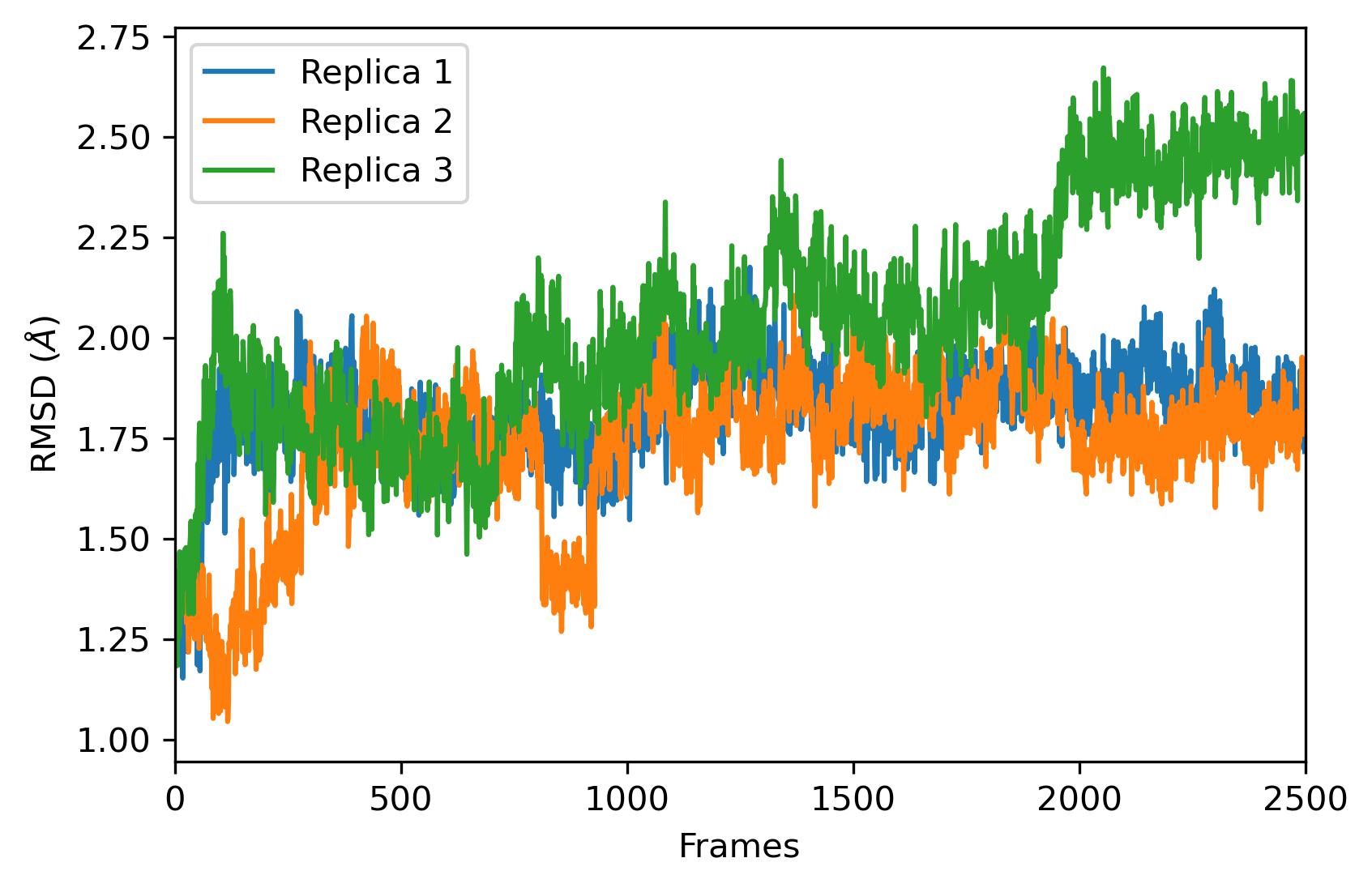


***SI Figure 6*** *RMSD of the atoms in the binding site from the crystallographic structure of the three HBI-ADBR2 trajectories.*

| **Molecule name** | **Activity type** | **Reference** | **Structure** |
| --- | --- | --- | --- |
| ABEDITEROL | AGONIST | [1] | 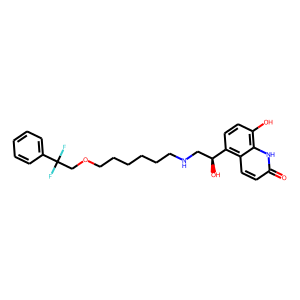 |
| BEDORADRINE | AGONIST | [1] | 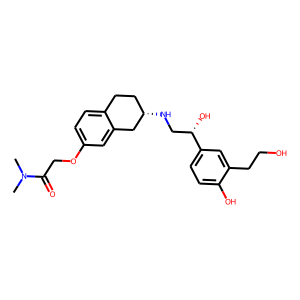 |
| CARMOTEROL | AGONIST | [1] | 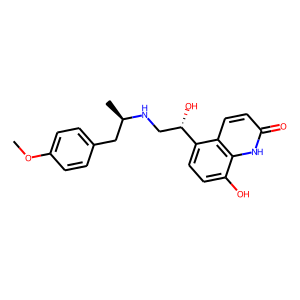 |
| CLENBUTEROL | AGONIST | [1] | 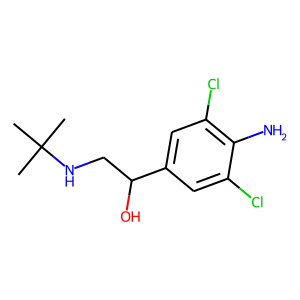 |
| DILEVALOL | AGONIST | [1] | 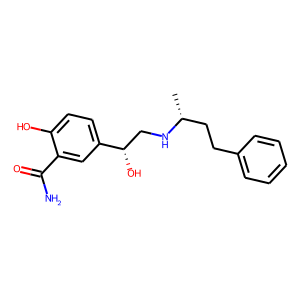 |
| DOBUTAMINE | AGONIST | [1] | 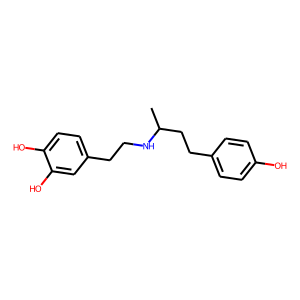 |
| FENOTEROL | AGONIST | [1] | 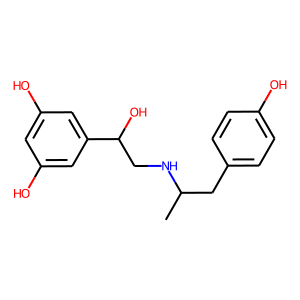 |
| INDACATEROL | AGONIST | [1] | 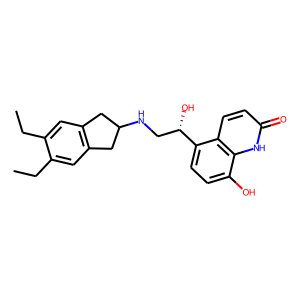 |
| ISOETHARINE | AGONIST | [1] | 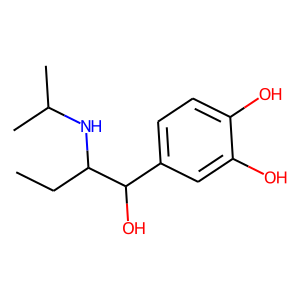 |
| ISOXSUPRINE | AGONIST | [1] | 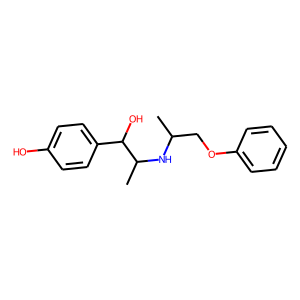 |
| MILVETEROL | AGONIST | [1] | 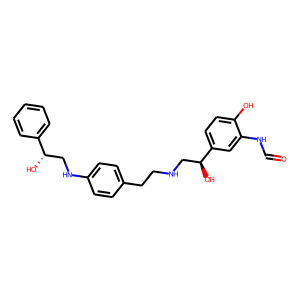 |
| OLODATEROL | AGONIST | [1] | 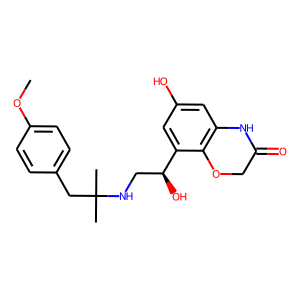 |
| PIRBUTEROL | AGONIST | [1] | 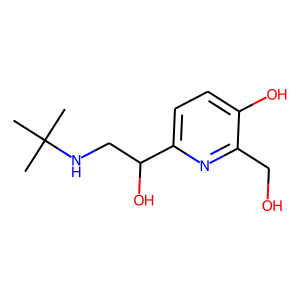 |
| PROCATEROL | AGONIST | [1] | 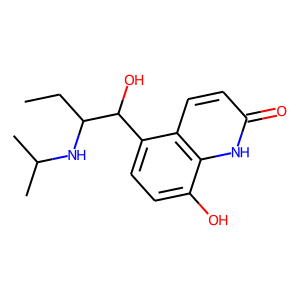 |
| PROTOKYLOL | AGONIST | [1] | 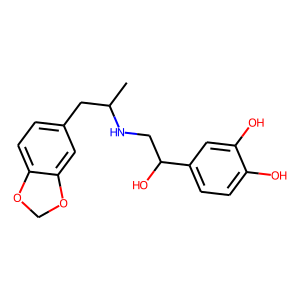 |
| RITODRINE | AGONIST | [1] | 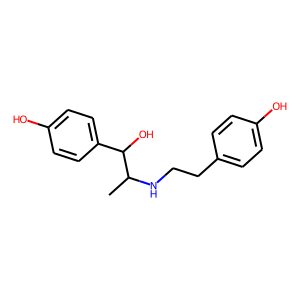 |
| SALMETEROL | AGONIST | [1] | 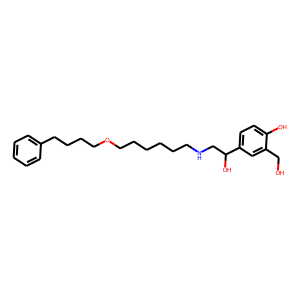 |
| TERBUTALINE | AGONIST | [1] | 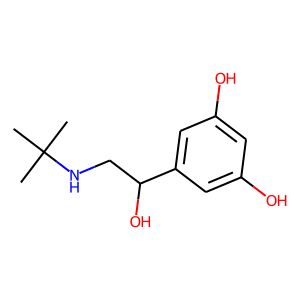 |
| TULOBUTEROL | AGONIST | [1] | 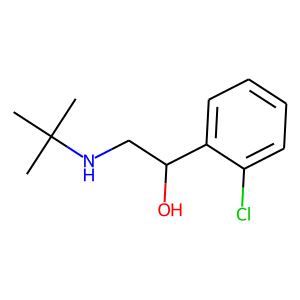 |
| 4 | ANTAGONIST | [2] | 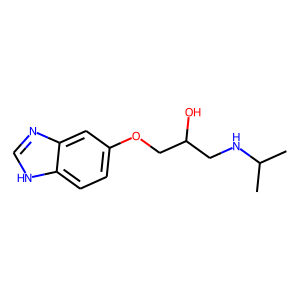 |
| 15a | ANTAGONIST | [3] | 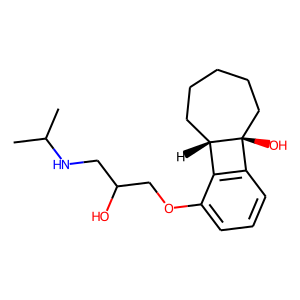 |
| BUPRANOLOL | ANTAGONIST | [4] | 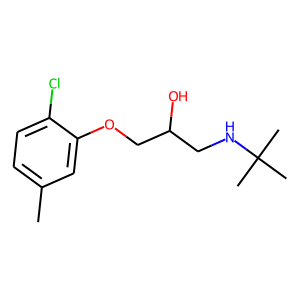 |
| BUTOXAMINE | ANTAGONIST | [5] | 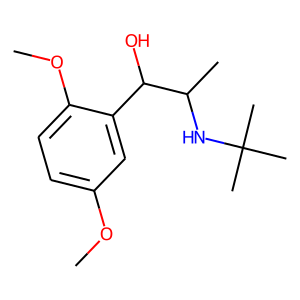 |
| CARVEDILOL | ANTAGONIST | [6] | 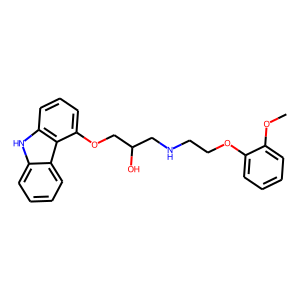 |
| ESMOLOL | ANTAGONIST | [1] | 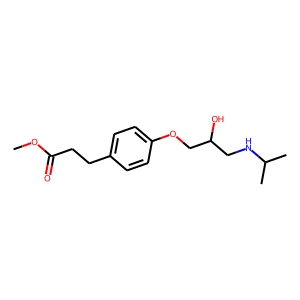 |
| ICI118551 | ANTAGONIST | [4] | 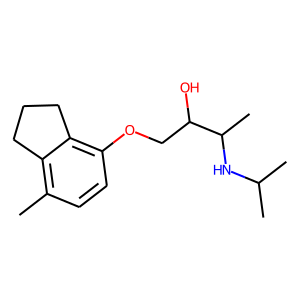 |
| IPS339 | ANTAGONIST | [4] | 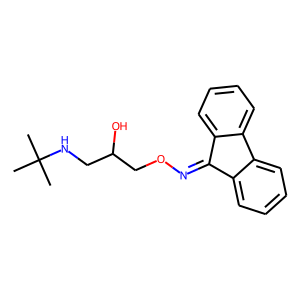 |
| BUNOLOL | ANTAGONIST | [1] | 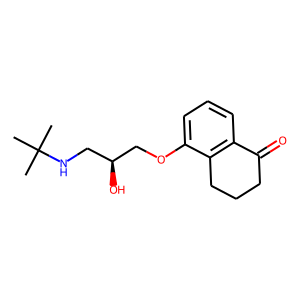 |
| METIPRANOLOL | ANTAGONIST | [1] | 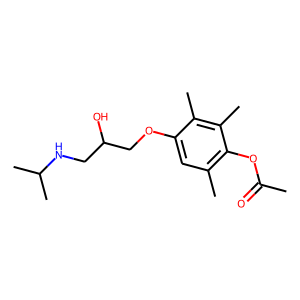 |
| NEBIVOLOL | ANTAGONIST | [1] | 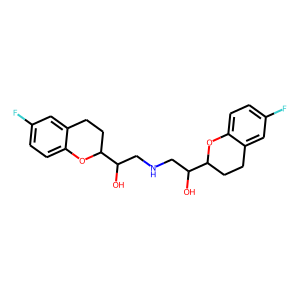 |
| PRONETALOL | ANTAGONIST | [1] | 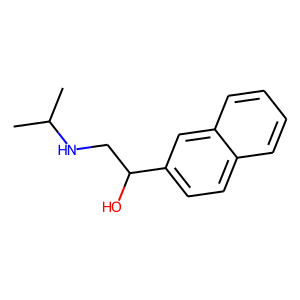 |
| PROPAFENONE | ANTAGONIST | [1] | 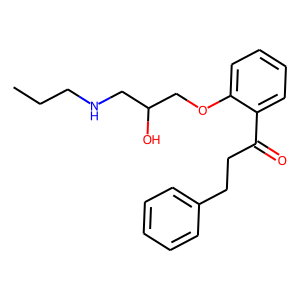 |
| PROPRANOLOL | ANTAGONIST | [1] | 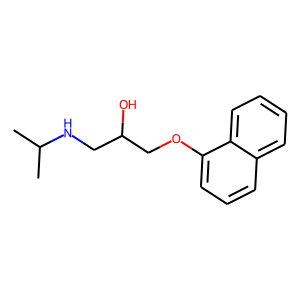 |
| SOTALOL | ANTAGONIST | [1] | 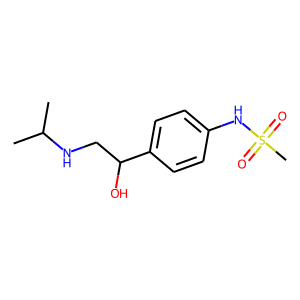 |
| SR59230A | ANTAGONIST | [5] | 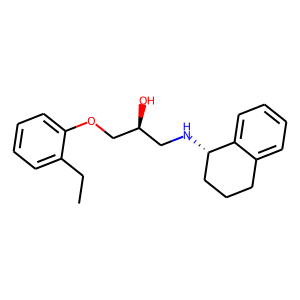 |
| TIMOLOL | ANTAGONIST | [1] | 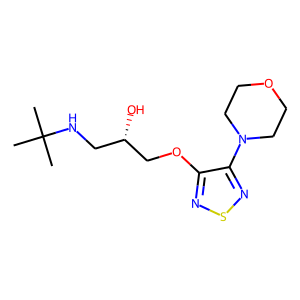 |

**SI Table 1** Agonist/antagonist *dataset containing ligands with known pharmacological activity.*

| **Molecule name** | **Activity type** | **Structure** |
| --- | --- | --- |
| 1 | AGONIST | 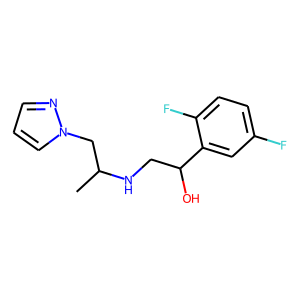 |
| 2 | AGONIST | 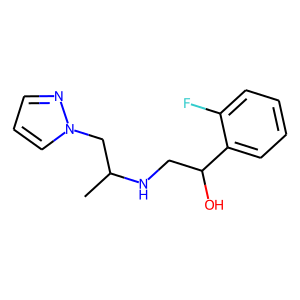 |
| 3 | AGONIST | 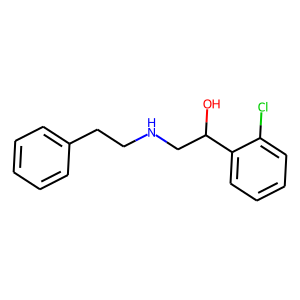 |
| 4 | AGONIST | 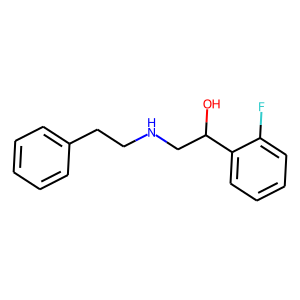 |
| 5 | AGONIST | 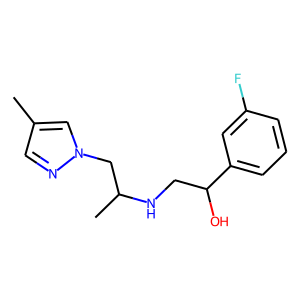 |
| 6 | AGONIST | 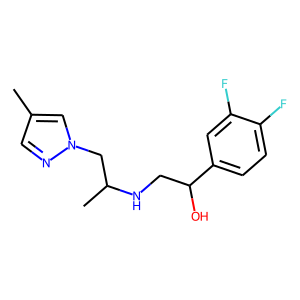 |
| 7 | AGONIST | 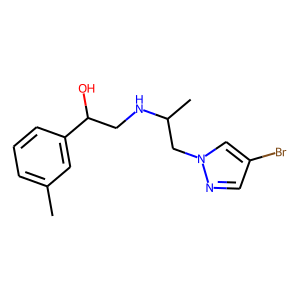 |
| 8 | AGONIST | 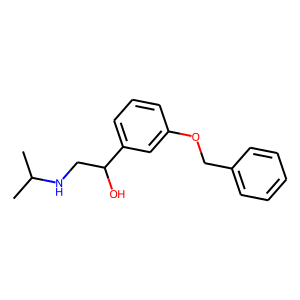 |
| 9 | INACTIVE | 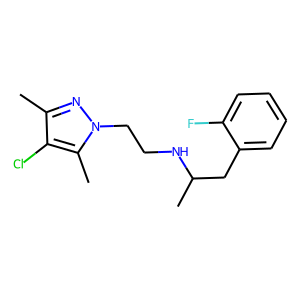 |
| 10 | INACTIVE | 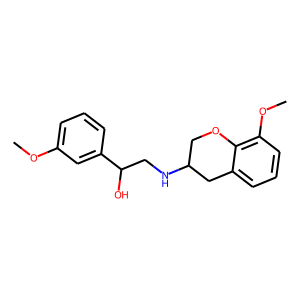 |
| 11 | AGONIST | 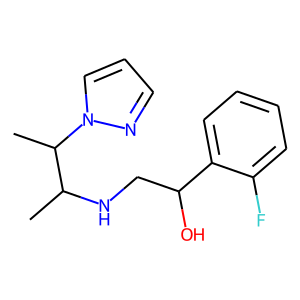 |
| 12 | AGONIST | 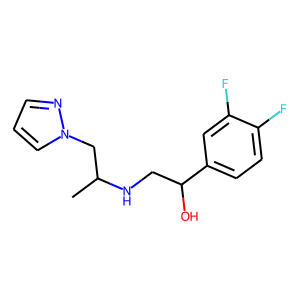 |
| 13 | INACTIVE | 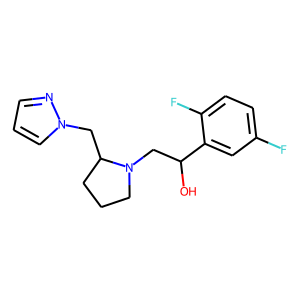 |
| 14 | INACTIVE | 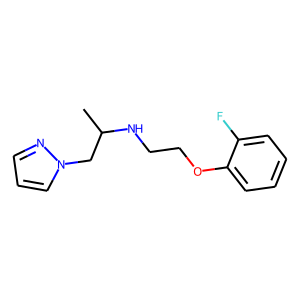 |
| 15 | INACTIVE | 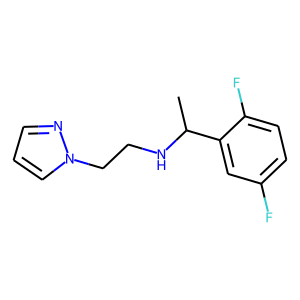 |
| 16 | INACTIVE | 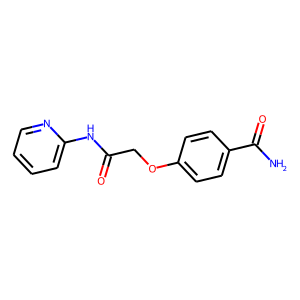 |
| 17 | INACTIVE | 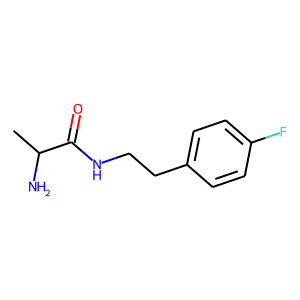 |
| 18 | INACTIVE | 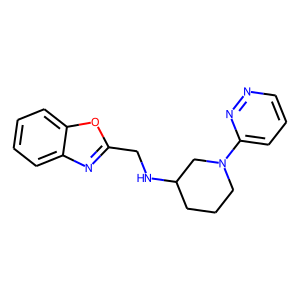 |
| 19 | INACTIVE | 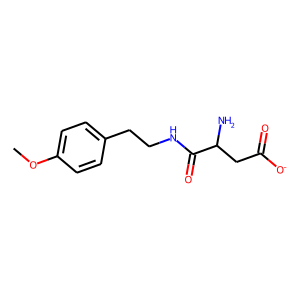 |
| 20 | INACTIVE | 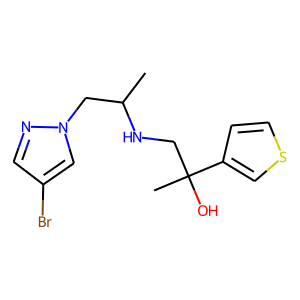 |
| 21 | INACTIVE | 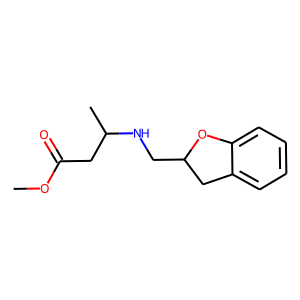 |
| 22 | INACTIVE | 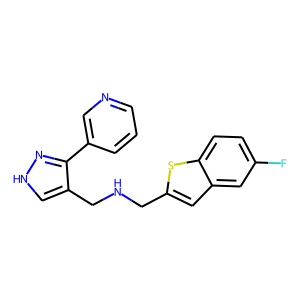 |
| 23 | INACTIVE | 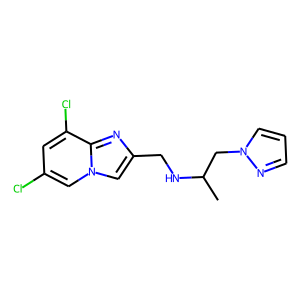 |
| 24 | INACTIVE | 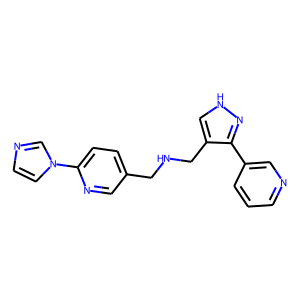 |
| 25 | INACTIVE | 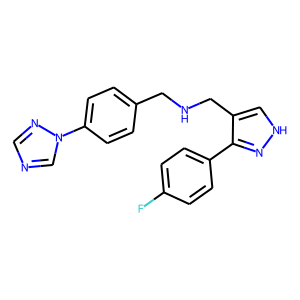 |
| 26 | INACTIVE | 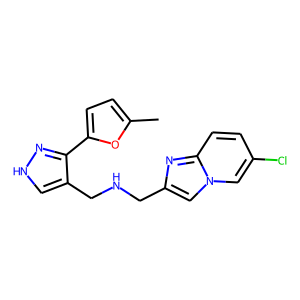 |
| 27 | INACTIVE | 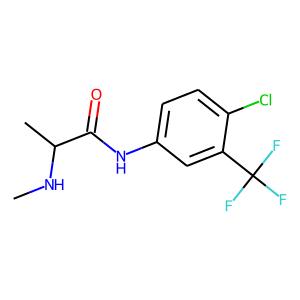 |

**SI Table 2** Agonist/inactive *dataset containing ligands with known activity*[7]*.*

| **Reference trajectory** | **Model** | **Precision** | | | **Recall** | | | **F1** | | |
| --- | --- | --- | --- | --- | --- | --- | --- | --- | --- | --- |
|  |  | **Avg** | **Max** | **Min** | **Avg** | **Max** | **Min** | **Avg** | **Max** | **Min** |
| Epinephrine | Hyd - MAD | 0.61 | 0.70 | 0.57 | 0.90 | 0.95 | 0.84 | 0.73 | 0.76 | 0.69 |
|  | Hyd – QMS2 | 0.61 | 0.68 | 0.58 | 0.89 | 0.95 | 0.79 | 0.72 | 0.74 | 0.71 |
|  | Hyd - NN | 0.53 | 0.53 | 0.53 | 1.00 | 1.00 | 1.00 | 0.69 | 0.69 | 0.69 |
|  | Newhyd - MAD | 0.58 | 0.67 | 0.53 | 0.94 | 1.00 | 0.84 | 0.72 | 0.79 | 0.69 |
|  | Newhyd – QMS2 | 0.60 | 0.67 | 0.54 | 0.94 | 1.00 | 0.84 | 0.74 | 0.79 | 0.70 |
|  | Newhyd - NN | 0.53 | 0.53 | 0.53 | 1.00 | 1.00 | 1.00 | 0.69 | 0.69 | 0.69 |
|  | Polar - MAD | 0.53 | 0.53 | 0.53 | 1.00 | 1.00 | 1.00 | 0.69 | 0.69 | 0.69 |
|  | Polar – QMS2 | 0.53 | 0.53 | 0.53 | 1.00 | 1.00 | 1.00 | 0.69 | 0.69 | 0.69 |
|  | Polar - NN | 0.53 | 0.53 | 0.53 | 1.00 | 1.00 | 1.000 | 0.69 | 0.69 | 0.69 |
| HBI | Hyd - MAD | 0.97 | 1.00 | 0.85 | 0.59 | 0.84 | 0.42 | 0.74 | 0.88 | 0.59 |
|  | Hyd – QMS2 | 0.93 | 1.00 | 0.78 | 0.53 | 0.79 | 0.37 | 0.68 | 0.83 | 0.50 |
|  | Hyd - NN | 0.53 | 0.54 | 0.53 | 1.00 | 1.00 | 1.00 | 0.69 | 0.70 | 0.69 |
|  | Newhyd - MAD | 0.87 | 0.94 | 0.80 | 0.69 | 0.89 | 0.47 | 0.77 | 0.91 | 0.60 |
|  | Newhyd – QMS2 | 0.89 | 1.00 | 0.78 | 0.55 | 0.84 | 0.26 | 0.68 | 0.86 | 0.41 |
|  | Newhyd - NN | 0.54 | 0.56 | 0.53 | 1.00 | 1.00 | 1.00 | 0.70 | 0.72 | 0.69 |
|  | Polar - MAD | 0.81 | 0.94 | 0.76 | 0.86 | 1.00 | 0.67 | 0.84 | 0.95 | 0.72 |
|  | Polar – QMS2 | 0.89 | 1.00 | 0.75 | 0.75 | 0.95 | 0.58 | 0.81 | 0.91 | 0.68 |
|  | Polar - NN | 0.87 | 0.94 | 0.77 | 0.82 | 0.95 | 0.58 | 0.85 | 0.92 | 0.71 |
| BI167107-A | Hyd - MAD | 1.00 | 1.00 | - | 0.09 | 0.26 | 0.00 | 0.16 | 0.41 | - |
|  | Hyd – QMS2 | 1.00 | 1.00 | - | 0.05 | 0.16 | 0.00 | 0.10 | 0.28 | - |
|  | Hyd - NN | 0.46 | 0.56 | 0.44 | 0.36 | 0.53 | 0.21 | 0.40 | 0.54 | 0.26 |
|  | Newhyd - MAD | 0.98 | 1.00 | 0.93 | 0.66 | 0.84 | 0.47 | 0.79 | 0.91 | 0.64 |
|  | Newhyd – QMS2 | 0.97 | 1.00 | 0.93 | 0.52 | 0.79 | 0.26 | 0.68 | 0.86 | 0.41 |
|  | Newhyd - NN | 0.56 | 0.59 | 0.53 | 1.00 | 1.00 | 1.00 | 0.71 | 0.74 | 0.69 |
|  | Polar - MAD | 1.00 | 1.00 | 1.00 | 0.40 | 0.63 | 0.32 | 0.57 | 0.77 | 0.48 |
|  | Polar – QMS2 | 1.00 | 1.00 | 1.00 | 0.37 | 0.63 | 0.05 | 0.65 | 0.77 | 0.10 |
|  | Polar - NN | 1.00 | 1.00 | 1.00 | 0.57 | 0.74 | 0.26 | 0.72 | 0.85 | 0.41 |
| BI167107-I | Hyd - MAD | 0.78 | 0.88 | 0.67 | 0.29 | 0.37 | 0.21 | 0.41 | 0.52 | 0.32 |
|  | Hyd – QMS2 | 0.59 | 0.67 | 0.50 | 0.18 | 0.21 | 0.16 | 0.28 | 0.32 | 0.24 |
|  | Hyd - NN | 0.55 | 0.58 | 0.53 | 0.92 | 1.00 | 0.84 | 0.69 | 0.73 | 0.65 |
|  | Newhyd - MAD | 0.66 | 0.74 | 0.58 | 0.66 | 0.74 | 0.58 | 0.66 | 0.58 | 0.74 |
|  | Newhyd – QMS2 | 0.58 | 0.60 | 0.55 | 0.60 | 0.63 | 0.58 | 0.59 | 0.61 | 0.56 |
|  | Newhyd - NN | 0.42 | 0.43 | 0.40 | 0.26 | 0.32 | 0.21 | 0.32 | 0.37 | 0.28 |
|  | Polar - MAD | 0.63 | 0.70 | 0.56 | 0.79 | 0.84 | 0.74 | 0.70 | 0.76 | 0.64 |
|  | Polar – QMS2 | 0.60 | 0.63 | 0.58 | 0.84 | 0.89 | 0.79 | 0.70 | 0.74 | 0.67 |
|  | Polar - NN | 0.78 | 0.78 | 0.78 | 0.95 | 0.95 | 0.95 | 0.86 | 0.86 | 0.86 |

**SI Table 3** Average performance of all OCSVM models on the agonist/antagonist dataset from docking data obtained using the representative structures. The average value/highest value/lowest value are reported for each set of parameters. Models discarding all docking poses are indicated as -.

| **Reference trajectory** | **Model** | **Precision** | **Recall** | **F1** |
| --- | --- | --- | --- | --- |
| Epinephrine | Hyd - MAD | 0.62 | 0.95 | 0.75 |
|  | Hyd – QMS2 | 0.58 | 0.95 | 0.72 |
|  | Hyd - NN | 0.53 | 1.00 | 0.69 |
|  | Newhyd - MAD | 0.53 | 1.00 | 0.69 |
|  | Newhyd – QMS2 | 0.54 | 1.00 | 0.70 |
|  | Newhyd - NN | 0.53 | 1.00 | 0.69 |
|  | Polar - MAD | 0.53 | 1.00 | 0.69 |
|  | Polar – QMS2 | 0.54 | 1.00 | 0.70 |
|  | Polar - NN | 0.53 | 1.00 | 0.69 |
| HBI | Hyd - MAD | 1.00 | 0.74 | 0.85 |
|  | Hyd – QMS2 | 1.00 | 0.74 | 0.85 |
|  | Hyd - NN | 0.54 | 1.00 | 0.70 |
|  | Newhyd - MAD | 1.00 | 0.68 | 0.80 |
|  | Newhyd – QMS2 | 1.00 | 0.42 | 0.59 |
|  | Newhyd - NN | 0.57 | 0.89 | 0.69 |
|  | Polar - MAD | 1.00 | 0.74 | 0.85 |
|  | Polar – QMS2 | 1.00 | 0.74 | 0.85 |
|  | Polar - NN | 0.94 | 0.79 | 0.86 |
| BI167107-A | Hyd - MAD | 1.00 | 0.47 | 0.64 |
|  | Hyd – QMS2 | 1.00 | 0.21 | 0.35 |
|  | Hyd - NN | 0.57 | 0.89 | 0.69 |
|  | Newhyd - MAD | 0.00 | 0.00 | - |
|  | Newhyd – QMS2 | - | 0.00 | - |
|  | Newhyd - NN | 0.44 | 0.37 | 0.40 |
|  | Polar - MAD | 1.00 | 0.21 | 0.35 |
|  | Polar – QMS2 | 1.00 | 0.32 | 0.48 |
|  | Polar - NN | 0.95 | 0.95 | 0.95 |

**SI Table 4** Performance of all OCSVM models on the agonist/antagonist dataset *from docking data obtained using crystallographic structures*. Models discarding all docking poses are indicated as -.

| **Reference trajectory** | **Model** | **Precision** | | | **Recall** | | | **F1** | | |
| --- | --- | --- | --- | --- | --- | --- | --- | --- | --- | --- |
|  |  | **Avg** | **Max** | **Min** | **Avg** | **Max** | **Min** | **Avg** | **Max** | **Min** |
| Epinephrine | Hyd - MAD | 0.40 | 0.42 | 0.36 | 1.00 | 1.00 | 1.00 | 0.56 | 0.59 | 0.53 |
|  | Hyd – QMS2 | 0.40 | 0.45 | 0.38 | 0.94 | 1.00 | 0.80 | 0.57 | 0.62 | 0.52 |
|  | Hyd - NN | 0.37 | 0.37 | 0.37 | 1.00 | 1.00 | 1.00 | 0.54 | 0.54 | 0.54 |
|  | Newhyd - MAD | 0.40 | 0.44 | 0.35 | 0.86 | 1.00 | 0.80 | 0.55 | 0.57 | 0.49 |
|  | Newhyd – QMS2 | 0.41 | 0.54 | 0.33 | 0.82 | 1.00 | 0.70 | 0.55 | 0.61 | 0.45 |
|  | Newhyd - NN | 0.37 | 0.37 | 0.37 | 1.00 | 1.00 | 1.00 | 0.54 | 0.54 | 0.54 |
|  | Polar - MAD | 0.40 | 0.43 | 0.37 | 1.00 | 1.00 | 1.00 | 0.57 | 0.60 | 0.54 |
|  | Polar – QMS2 | 0.40 | 0.43 | 0.38 | 1.00 | 1.00 | 1.00 | 0.57 | 0.60 | 0.55 |
|  | Polar - NN | 0.37 | 0.37 | 0.37 | 1.00 | 1.00 | 1.00 | 0.54 | 0.54 | 0.54 |
| HBI | Hyd - MAD | 0.71 | 1.00 | - | 0.17 | 0.60 | 0.00 | 0.27 | 0.75 | - |
|  | Hyd – QMS2 | 0.71 | 1.00 | 0.00 | 0.12 | 0.40 | 0.00 | 0.20 | 0.57 | 0.00 |
|  | Hyd - NN | 0.45 | 0.50 | 0.42 | 1.00 | 1.00 | 1.00 | 0.62 | 0.65 | 0.59 |
|  | Newhyd - MAD | 1.00 | 1.00 | - | 0.23 | 0.50 | 0.00 | 0.37 | 0.67 | - |
|  | Newhyd – QMS2 | 1.00 | 1.00 | - | 0.23 | 0.60 | 0.00 | 0.37 | 0.67 | - |
|  | Newhyd - NN | 0.40 | 0.40 | 0.38 | 1.00 | 1.00 | 1.00 | 0.57 | 0.57 | 0.55 |
|  | Polar - MAD | 0.57 | 1.00 | 0.00 | 0.08 | 0.50 | 0.00 | 0.14 | 0.62 | 0.00 |
|  | Polar – QMS2 | 1.00 | 1.00 | - | 0.08 | 0.50 | 0.00 | 0.15 | 0.67 | - |
|  | Polar - NN | 0.26 | 0.86 | 0.00 | 0.08 | 0.60 | 0.00 | 0.12 | 0.71 | 0.00 |
| BI167107-A | Hyd - MAD | 0.10 | 0.10 | - | 0.02 | 0.10 | 0.00 | 0.04 | 0.10 | - |
|  | Hyd – QMS2 | - | - | - | 0.00 | 0.00 | 0.00 | - | - | - |
|  | Hyd - NN | 0.56 | 0.58 | 0.54 | 0.72 | 0.90 | 0.60 | 0.63 | 0.69 | 0.57 |
|  | Newhyd - MAD | 1.00 | 1.00 | - | 0.08 | 0.30 | 0.00 | 0.14 | 0.46 | - |
|  | Newhyd – QMS2 | 1.00 | 1.00 | - | 0.05 | 0.20 | 0.00 | 0.10 | 0.33 | - |
|  | Newhyd - NN | 0.44 | 0.45 | 0.43 | 1.00 | 1.00 | 1.00 | 0.61 | 0.62 | 0.60 |
|  | Polar - MAD | - | - | - | 0.00 | 0.00 | 0.00 | - | - | - |
|  | Polar – QMS2 | - | - | - | 0.00 | 0.00 | 0.00 | - | - | - |
|  | Polar - NN | - | - | - | 0.00 | 0.00 | 0.00 | - | - | - |
| BI167107-I | Hyd - MAD | 0.00 | - | 0.00 | 0.00 | 0.00 | 0.00 | 0.00 | - | 0.00 |
|  | Hyd – QMS2 | 0.00 | - | 0.00 | 0.00 | 0.00 | 0.00 | 0.00 | - | 0.00 |
|  | Hyd - NN | 0.58 | 0.62 | 0.55 | 0.70 | 0.80 | 0.60 | 0.64 | 0.70 | 0.57 |
|  | Newhyd - MAD | 0.79 | 0.83 | 0.75 | 0.55 | 0.60 | 0.50 | 0.65 | 0.67 | 0.62 |
|  | Newhyd – QMS2 | 0.73 | 0.75 | 0.71 | 0.55 | 0.60 | 0.50 | 0.63 | 0.67 | 0.59 |
|  | Newhyd - NN | 0.58 | 0.62 | 0.55 | 0.70 | 0.80 | 0.60 | 0.64 | 0.70 | 0.57 |
|  | Polar - MAD | 0.52 | 0.53 | 0.50 | 0.55 | 0.90 | 0.20 | 0.53 | 0.66 | 0.29 |
|  | Polar – QMS2 | 0.48 | 0.56 | 0.40 | 0.60 | 1.00 | 0.20 | 0.53 | 0.72 | 0.28 |
|  | Polar - NN | 0.80 | 0.86 | 0.75 | 0.60 | 0.60 | 0.60 | 0.69 | 0.71 | 0.67 |

**SI Table 5** Average performance of all OCSVM models on the agonist/inactive dataset from docking data obtained using the representative structures. The average value/highest value/lowest value are reported for each set of parameters. Models discarding all docking poses are indicated as -.

| **Reference trajectory** | **Model** | **Precision** | **Recall** | **F1** |
| --- | --- | --- | --- | --- |
| Epinephrine | Hyd - MAD | 0.38 | 1.00 | 0.56 |
|  | Hyd – QMS2 | 0.39 | 0.90 | 0.55 |
|  | Hyd - NN | 0.37 | 1.00 | 0.54 |
|  | Newhyd - MAD | 0.38 | 1.00 | 0.56 |
|  | Newhyd – QMS2 | 0.40 | 1.00 | 0.57 |
|  | Newhyd - NN | 0.37 | 1.00 | 0.54 |
|  | Polar - MAD | 0.42 | 1.00 | 0.59 |
|  | Polar – QMS2 | 0.40 | 1.00 | 0.57 |
|  | Polar - NN | 0.37 | 1.00 | 0.54 |
| HBI | Hyd - MAD | 1.00 | 0.70 | 0.82 |
|  | Hyd – QMS2 | 1.00 | 0.30 | 0.46 |
|  | Hyd - NN | 0.50 | 1.00 | 0.67 |
|  | Newhyd - MAD | 1.00 | 0.50 | 0.67 |
|  | Newhyd – QMS2 | 1.00 | 0.60 | 0.75 |
|  | Newhyd - NN | 0.42 | 1.00 | 0.59 |
|  | Polar - MAD | - | 0.00 | - |
|  | Polar – QMS2 | - | 0.00 | - |
|  | Polar - NN | 1.00 | 0.10 | 0.18 |
| BI167107-A | Hyd - MAD | 0.00 | 0.00 | 0.00 |
|  | Hyd – QMS2 | 0.00 | 0.00 | 0.00 |
|  | Hyd - NN | 0.50 | 0.60 | 0.55 |
|  | Newhyd - MAD | 0.00 | 0.00 | 0.00 |
|  | Newhyd – QMS2 | 0.00 | 0.00 | 0.00 |
|  | Newhyd - NN | 0.43 | 1.00 | 0.61 |
|  | Polar - MAD | - | 0.00 | - |
|  | Polar – QMS2 | - | 0.00 | - |
|  | Polar - NN | - | 0.00 | - |

**SI Table 6** Performances of all OCSVM models on the agonist/inactive dataset *from docking data obtained using crystallographic structures*. Models discarding all docking poses are indicated as -.

| **Reference trajectory** | **Model** | **Precision** | | | **Recall** | | | **F1** | | |
| --- | --- | --- | --- | --- | --- | --- | --- | --- | --- | --- |
|  |  | **Avg** | **Max** | **Min** | **Avg** | **Max** | **Min** | **Avg** | **Max** | **Min** |
| Epinephrine 25% | Hyd - MAD | 0.64 | 0.72 | 0.53 | 0.91 | 1.00 | 0.84 | 0.75 | 0.82 | 0.68 |
|  | Hyd – QMS2 | 0.62 | 0.68 | 0.53 | 0.92 | 1.00 | 0.84 | 0.74 | 0.79 | 0.68 |
|  | Hyd - NN | 0.53 | 0.53 | 0.53 | 1.00 | 1.00 | 1.00 | 0.69 | 0.69 | 0.69 |
|  | Newhyd - MAD | 0.64 | 0.72 | 0.53 | 0.91 | 1.00 | 0.84 | 0.75 | 0.82 | 0.68 |
|  | Newhyd – QMS2 | 0.62 | 0.68 | 0.53 | 0.92 | 1.00 | 0.84 | 0.74 | 0.79 | 0.68 |
|  | Newhyd - NN | 0.53 | 0.53 | 0.53 | 1.00 | 1.00 | 1.00 | 0.69 | 0.69 | 0.69 |
|  | Polar - MAD | 0.53 | 0.53 | 0.53 | 1.00 | 1.00 | 1.00 | 0.69 | 0.69 | 0.69 |
|  | Polar – QMS2 | 0.54 | 0.59 | 0.51 | 0.99 | 1.00 | 0.95 | 0.70 | 0.74 | 0.66 |
|  | Polar - NN | 0.53 | 0.53 | 0.53 | 1.00 | 1.00 | 1.00 | 0.69 | 0.69 | 0.69 |
| Epinephrine 50% | Hyd - MAD | 0.63 | 0.75 | 0.53 | 0.91 | 1.00 | 0.84 | 0.75 | 0.84 | 0.68 |
|  | Hyd – QMS2 | 0.63 | 0.68 | 0.54 | 0.92 | 1.00 | 0.89 | 0.75 | 0.79 | 0.70 |
|  | Hyd - NN | 0.53 | 0.53 | 0.53 | 1.00 | 1.00 | 1.00 | 0.69 | 0.69 | 0.69 |
|  | Newhyd - MAD | 0.63 | 0.75 | 0.53 | 0.91 | 1.00 | 0.84 | 0.75 | 0.84 | 0.68 |
|  | Newhyd – QMS2 | 0.63 | 0.68 | 0.54 | 0.92 | 1.00 | 0.84 | 0.75 | 0.79 | 0.70 |
|  | Newhyd - NN | 0.53 | 0.53 | 0.53 | 1.00 | 1.00 | 1.00 | 0.69 | 0.69 | 0.69 |
|  | Polar - MAD | 0.53 | 0.53 | 0.53 | 1.00 | 1.00 | 1.00 | 0.69 | 0.69 | 0.69 |
|  | Polar – QMS2 | 0.53 | 0.54 | 0.53 | 1.00 | 1.00 | 1.00 | 0.69 | 0.70 | 0.69 |
|  | Polar - NN | 0.53 | 0.53 | 0.53 | 1.00 | 1.00 | 1.00 | 0.69 | 0.69 | 0.69 |
| Epinephrine 75% | Hyd - MAD | 0.59 | 0.69 | 0.51 | 0.93 | 1.00 | 0.84 | 0.72 | 0.84 | 0.68 |
|  | Hyd – QMS2 | 0.61 | 0.69 | 0.54 | 0.93 | 1.00 | 0.89 | 0.73 | 0.80 | 0.70 |
|  | Hyd - NN | 0.53 | 0.53 | 0.53 | 1.00 | 1.00 | 1.00 | 0.69 | 0.69 | 0.69 |
|  | Newhyd - MAD | 0.59 | 0.69 | 0.51 | 0.93 | 1.00 | 0.84 | 0.72 | 0.80 | 0.66 |
|  | Newhyd – QMS2 | 0.61 | 0.69 | 0.54 | 0.93 | 1.00 | 0.84 | 0.74 | 0.80 | 0.70 |
|  | Newhyd - NN | 0.53 | 0.53 | 0.53 | 1.00 | 1.00 | 1.00 | 0.69 | 0.69 | 0.69 |
|  | Polar - MAD | 0.53 | 0.53 | 0.53 | 1.00 | 1.00 | 1.00 | 0.69 | 0.69 | 0.69 |
|  | Polar – QMS2 | 0.53 | 0.53 | 0.53 | 1.00 | 1.00 | 1.00 | 0.69 | 0.69 | 0.69 |
|  | Polar - NN | 0.53 | 0.53 | 0.53 | 1.00 | 1.00 | 1.00 | 0.69 | 0.69 | 0.69 |

**SI Table 7** Average performance on the agonist/antagonist dataset of the Epinephrine models trained using different percentages of the frames from docking data obtained using the representative structures. The average value/highest value/lowest value are reported for each set of parameters.

| **Reference trajectory** | **Model** | **Precision** | **Recall** | **F1** |
| --- | --- | --- | --- | --- |
| Epinephrine 25% | Hyd - MAD | 0.77 | 0.89 | 0.82 |
|  | Hyd – QMS2 | 0.76 | 0.84 | 0.80 |
|  | Hyd - NN | 0.53 | 1.00 | 0.69 |
|  | Newhyd - MAD | 0.53 | 1.00 | 0.69 |
|  | Newhyd – QMS2 | 0.54 | 1.00 | 0.70 |
|  | Newhyd - NN | 0.53 | 1.00 | 0.69 |
|  | Polar - MAD | 0.53 | 1.00 | 0.69 |
|  | Polar – QMS2 | 0.56 | 1.00 | 0.72 |
|  | Polar - NN | 0.53 | 1.00 | 0.69 |
| Epinephrine 50% | Hyd - MAD | 0.77 | 0.89 | 0.82 |
|  | Hyd – QMS2 | 0.70 | 0.89 | 0.78 |
|  | Hyd - NN | 0.53 | 1.00 | 0.69 |
|  | Newhyd - MAD | 0.54 | 1.00 | 0.70 |
|  | Newhyd – QMS2 | 0.56 | 1.00 | 0.72 |
|  | Newhyd - NN | 0.53 | 1.00 | 0.69 |
|  | Polar - MAD | 0.53 | 1.00 | 0.69 |
|  | Polar – QMS2 | 0.54 | 1.00 | 0.70 |
|  | Polar - NN | 0.53 | 1.00 | 0.69 |
| Epinephrine 75% | Hyd - MAD | 0.62 | 0.95 | 0.75 |
|  | Hyd – QMS2 | 0.58 | 0.95 | 0.72 |
|  | Hyd - NN | 0.53 | 1.00 | 0.69 |
|  | Newhyd - MAD | 0.53 | 1.00 | 0.69 |
|  | Newhyd – QMS2 | 0.56 | 1.00 | 0.72 |
|  | Newhyd - NN | 0.53 | 1.00 | 0.69 |
|  | Polar - MAD | 0.53 | 1.00 | 0.69 |
|  | Polar – QMS2 | 0.54 | 1.00 | 0.70 |
|  | Polar - NN | 0.53 | 1.00 | 0.69 |

**SI Table 8** Performance on the agonist/antagonist dataset *from docking data obtained using crystallographic structures* of the Epinephrine models trained using different percentages of the frames.

| **Reference trajectory** | **Model** | **Precision** | | | **Recall** | | | **F1** | | |
| --- | --- | --- | --- | --- | --- | --- | --- | --- | --- | --- |
|  |  | **Avg** | **Max** | **Min** | **Avg** | **Max** | **Min** | **Avg** | **Max** | **Min** |
| HBI 25% | Hyd - MAD | 1.00 | 1.00 | 1.00 | 0.43 | 0.84 | 0.21 | 0.60 | 0.91 | 0.35 |
|  | Hyd – QMS2 | 1.00 | 1.00 | 1.00 | 0.40 | 0.58 | 0.26 | 0.58 | 0.73 | 0.41 |
|  | Hyd - NN | 0.53 | 0.53 | 0.53 | 1.00 | 1.00 | 1.00 | 0.69 | 0.69 | 0.69 |
|  | Newhyd - MAD | 0.85 | 0.94 | 0.75 | 0.51 | 0.79 | 0.32 | 0.64 | 0.86 | 0.47 |
|  | Newhyd – QMS2 | 0.98 | 1.00 | - | 0.31 | 0.42 | 0.00 | 0.47 | 0.59 | - |
|  | Newhyd - NN | 0.53 | 0.53 | 0.53 | 1.00 | 1.00 | 1.00 | 0.69 | 0.69 | 0.69 |
|  | Polar - MAD | 0.92 | 1.00 | 0.80 | 0.63 | 0.79 | 0.26 | 0.75 | 0.88 | 0.41 |
|  | Polar – QMS2 | 0.92 | 1.00 | 0.79 | 0.54 | 0.84 | 0.11 | 0.68 | 0.82 | 0.20 |
|  | Polar - NN | 0.90 | 1.00 | 0.75 | 0.70 | 0.84 | 0.37 | 0.78 | 0.86 | 0.54 |
| HBI 50% | Hyd - MAD | 1.00 | 1.00 | 1.00 | 0.36 | 0.74 | 0.16 | 0.53 | 0.85 | 0.20 |
|  | Hyd – QMS2 | 1.00 | 1.00 | 1.00 | 0.43 | 0.68 | 0.26 | 0.60 | 0.81 | 0.41 |
|  | Hyd - NN | 0.53 | 0.53 | 0.53 | 1.00 | 1.00 | 1.00 | 0.69 | 0.69 | 0.69 |
|  | Newhyd - MAD | 0.97 | 1.00 | 0.88 | 0.44 | 0.79 | 0.21 | 0.61 | 0.88 | 0.35 |
|  | Newhyd – QMS2 | 1.00 | 1.00 | - | 0.32 | 0.47 | 0.00 | 0.48 | 0.64 | - |
|  | Newhyd - NN | 0.54 | 0.59 | 0.53 | 1.00 | 1.00 | 1.00 | 0.70 | 0.74 | 0.69 |
|  | Polar - MAD | 0.92 | 1.00 | 0.80 | 0.65 | 0.79 | 0.37 | 0.76 | 0.88 | 0.54 |
|  | Polar – QMS2 | 0.92 | 1.00 | 0.77 | 0.56 | 0.84 | 0.26 | 0.70 | 0.85 | 0.41 |
|  | Polar - NN | 0.90 | 1.00 | 0.75 | 0.67 | 0.84 | 0.37 | 0.77 | 0.86 | 0.54 |
| HBI 75% | Hyd - MAD | 0.99 | 1.00 | 0.89 | 0.48 | 0.84 | 0.26 | 0.64 | 0.91 | 0.41 |
|  | Hyd – QMS2 | 0.98 | 1.00 | 0.88 | 0.49 | 0.74 | 0.32 | 0.65 | 0.80 | 0.48 |
|  | Hyd - NN | 0.53 | 0.53 | 0.53 | 1.00 | 1.00 | 1.00 | 0.69 | 0.69 | 0.69 |
|  | Newhyd - MAD | 0.87 | 0.94 | 0.75 | 0.52 | 0.79 | 0.32 | 0.65 | 0.86 | 0.45 |
|  | Newhyd – QMS2 | 0.97 | 1.00 | 0.83 | 0.37 | 0.53 | 0.05 | 0.53 | 0.69 | 0.10 |
|  | Newhyd - NN | 0.53 | 0.56 | 0.51 | 1.00 | 1.00 | 0.95 | 0.69 | 0.72 | 0.66 |
|  | Polar - MAD | 0.88 | 1.00 | 0.71 | 0.67 | 0.89 | 0.37 | 0.76 | 0.91 | 0.54 |
|  | Polar – QMS2 | 0.89 | 1.00 | 0.69 | 0.62 | 0.95 | 0.32 | 0.73 | 0.88 | 0.56 |
|  | Polar - NN | 0.92 | 1.00 | 0.75 | 0.68 | 0.84 | 0.37 | 0.78 | 0.86 | 0.54 |

**SI Table 9** Average performance on the agonist/antagonist dataset of the HBI models trained using different percentages of the frames from docking data obtained using the representative structures. The average value/highest value/lowest value are reported for each set of parameters. Models discarding all docking poses are indicated as -.

| **Reference trajectory** | **Model** | **Precision** | **Recall** | **F1** |
| --- | --- | --- | --- | --- |
| HBI 25% | Hyd - MAD | 1.00 | 0.58 | 0.73 |
|  | Hyd – QMS2 | 1.00 | 0.53 | 0.69 |
|  | Hyd - NN | 0.54 | 1.00 | 0.70 |
|  | Newhyd - MAD | 1.00 | 0.58 | 0.73 |
|  | Newhyd – QMS2 | 1.00 | 0.11 | 0.20 |
|  | Newhyd - NN | 0.57 | 0.84 | 0.68 |
|  | Polar - MAD | 1.00 | 0.63 | 0.77 |
|  | Polar – QMS2 | 1.00 | 0.63 | 0.77 |
|  | Polar - NN | 1.00 | 0.58 | 0.73 |
| HBI 50% | Hyd - MAD | 1.00 | 0.53 | 0.69 |
|  | Hyd – QMS2 | 1.00 | 0.53 | 0.69 |
|  | Hyd - NN | 0.54 | 1.00 | 0.70 |
|  | Newhyd - MAD | 1.00 | 0.53 | 0.69 |
|  | Newhyd – QMS2 | 1.00 | 0.21 | 0.35 |
|  | Newhyd - NN | 0.57 | 0.84 | 0.68 |
|  | Polar - MAD | 1.00 | 0.63 | 0.77 |
|  | Polar – QMS2 | 1.00 | 0.63 | 0.77 |
|  | Polar - NN | 1.00 | 0.47 | 0.64 |
| HBI 75% | Hyd - MAD | 1.00 | 0.42 | 0.59 |
|  | Hyd – QMS2 | 1.00 | 0.58 | 0.73 |
|  | Hyd - NN | 0.54 | 1.00 | 0.70 |
|  | Newhyd - MAD | 1.00 | 0.58 | 0.73 |
|  | Newhyd – QMS2 | 1.00 | 0.21 | 0.35 |
|  | Newhyd - NN | 0.57 | 0.89 | 0.69 |
|  | Polar - MAD | 1.00 | 0.63 | 0.77 |
|  | Polar – QMS2 | 1.00 | 0.58 | 0.73 |
|  | Polar - NN | 1.00 | 0.47 | 0.64 |

**SI Table 10** Performance on the agonist/antagonist dataset *from docking data obtained using crystallographic structures* of the HBI models using different percentages of the frames.

| **Ligand** | **Model** | **Precision** | | | **Recall** | | | **F1** | | |
| --- | --- | --- | --- | --- | --- | --- | --- | --- | --- | --- |
|  |  | **Avg** | **Max** | **Min** | **Avg** | **Max** | **Min** | **Avg** | **Max** | **Min** |
| All | Hyd - MAD | 0.70 | 0.84 | 0.64 | 0.85 | 0.95 | 0.63 | 0.77 | 0.85 | 0.68 |
|  | Hyd – QMS2 | 0.57 | 0.62 | 0.52 | 0.97 | 1.00 | 0.89 | 0.72 | 0.76 | 0.66 |
|  | Newhyd - MAD | 0.64 | 0.75 | 0.59 | 0.92 | 1.00 | 0.79 | 0.75 | 0.82 | 0.69 |
|  | Newhyd – QMS2 | 0.55 | 0.58 | 0.53 | 1.00 | 1.00 | 0.95 | 0.71 | 0.73 | 0.69 |
|  | Polar - MAD | 0.53 | 0.53 | 0.53 | 1.00 | 1.00 | 1.00 | 0.69 | 0.69 | 0.69 |
|  | Polar – QMS2 | 0.63 | 0.76 | 0.50 | 0.93 | 1.00 | 0.63 | 0.75 | 0.86 | 0.65 |
|  | Polar - NN | 0.53 | 0.53 | 0.53 | 1.00 | 1.00 | 1.00 | 0.69 | 0.69 | 0.69 |
| BI167107-A & HBI | Hyd - MAD | 0.86 | 1.00 | 0.71 | 0.72 | 0.84 | 0.47 | 0.78 | 0.89 | 0.64 |
|  | Hyd – QMS2 | 0.85 | 0.94 | 0.71 | 0.61 | 0.84 | 0.42 | 0.71 | 0.91 | 0.57 |
|  | Newhyd - MAD | 0.84 | 0.95 | 0.78 | 0.78 | 0.95 | 0.58 | 0.81 | 0.95 | 0.69 |
|  | Newhyd – QMS2 | 0.83 | 0.95 | 0.70 | 0.65 | 0.95 | 0.53 | 0.73 | 0.95 | 0.63 |
|  | Polar - MAD | 0.89 | 1.00 | 0.76 | 0.82 | 1.00 | 0.68 | 0.85 | 0.95 | 0.72 |
|  | Polar – QMS2 | 0.93 | 1.00 | 0.80 | 0.74 | 0.89 | 0.58 | 0.82 | 0.91 | 0.69 |
|  | Polar - NN | 0.91 | 1.00 | 0.77 | 0.75 | 0.89 | 0.42 | 0.82 | 0.89 | 0.59 |

**SI Table 11** Average performance on the agonist/antagonist dataset of the models using multiple trajectories as reference for the training set from docking data obtained using the representative structures. The average value/highest value/lowest value are reported for each set of parameters.

| **Ligand** | **Model** | **Precision** | **Recall** | **F1** |
| --- | --- | --- | --- | --- |
| All | Hyd - MAD | 0.68 | 0.87 | 0.76 |
|  | Hyd – QMS2 | 0.58 | 1.00 | 0.73 |
|  | Newhyd - MAD | 0.58 | 1.00 | 0.73 |
|  | Newhyd – QMS2 | 0.54 | 1.00 | 0.70 |
|  | Polar - MAD | 0.57 | 0.88 | 0.70 |
|  | Polar – QMS2 | 0.63 | 0.97 | 0.76 |
|  | Polar - NN | 0.53 | 1.00 | 0.69 |
| BI167107-A & HBI | Hyd - MAD | 0.88 | 0.90 | 0.89 |
|  | Hyd – QMS2 | 0.94 | 0.84 | 0.89 |
|  | Newhyd - MAD | 0.92 | 0.68 | 0.78 |
|  | Newhyd – QMS2 | 0.88 | 0.42 | 0.57 |
|  | Polar - MAD | 1.00 | 0.74 | 0.85 |
|  | Polar – QMS2 | 1.00 | 0.71 | 0.83 |
|  | Polar - NN | 0.92 | 0.87 | 0.89 |

**SI Table 12** Performance on the agonist/antagonist dataset *from docking data obtained using crystallographic structures* of the models using multiple trajectories as reference for the training set.

| **Ligand** | **Threshold** | **Precision** | | | **Recall** | | |
| --- | --- | --- | --- | --- | --- | --- | --- |
|  |  | **Avg** | **Max** | **Min** | **Avg** | **Max** | **Min** |
| Epinephrine | 0.59 | 0.55 | 0.59 | 0.52 | 0.97 | 1.00 | 0.89 |
|  | 0.65 | 0.70 | 0.78 | 0.53 | 0.62 | 0.89 | 0.11 |
|  | 0.70 | 0.84 | 1.00 | - | 0.30 | 0.74 | 0.00 |
|  | 1.00 | - | - | - | 0.00 | 0.00 | 0.00 |
| HBI | 0.59 | 0.53 | 0.56 | 0.53 | 1.00 | 1.00 | 1.00 |
|  | 0.65 | 0.60 | 0.70 | 0.54 | 0.99 | 1.00 | 0.95 |
|  | 0.70 | 0.67 | 0.82 | 0.59 | 0.98 | 1.00 | 0.95 |
|  | 1.00 | 1.00 | 1.00 | - | 0.10 | 0.32 | 0.00 |
| BI167107 active | 0.59 | 0.53 | 0.53 | 0.53 | 1.00 | 1.00 | 1.00 |
|  | 0.65 | 0.58 | 0.59 | 0.54 | 1.00 | 1.00 | 1.00 |
|  | 0.70 | 0.64 | 0.57 | 0.61 | 0.99 | 1.00 | 0.95 |
|  | 1.00 | 1.00 | 1.00 | - | 0.10 | 0.16 | 0.00 |
| BI167107 inactive | 0.59 | 0.53 | 0.53 | 0.53 | 1.00 | 1.00 | 1.00 |
|  | 0.65 | 0.61 | 0.61 | 0.61 | 0.94 | 1.00 | 0.89 |
|  | 0.70 | 0.69 | 0.70 | 0.68 | 0.84 | 1.00 | 0.68 |
|  | 1.00 | 1.00 | 1.00 | - | 0.02 | 0.05 | 0.00 |

***SI Table 13*** Average *GRIM results on the agonists-antagonist dataset* from docking data obtained using the representative structures*. The standard definition of hydrophobic contacts is applied.* The average value/highest value/lowest value are reported for each threshold. *Thresholds discarding all docking poses are indicated as -.*

| **Ligand** | **Threshold** | **Precision** | **Recall** |
| --- | --- | --- | --- |
| Epinephrine | 0.59 | 0.53 | 1.00 |
|  | 0.65 | 0.63 | 1.00 |
|  | 0.70 | 0.86 | 1.00 |
|  | 1.00 | - | 0.00 |
| HBI | 0.59 | 0.53 | 1.00 |
|  | 0.65 | 0.59 | 1.00 |
|  | 0.70 | 0.68 | 1.00 |
|  | 1.00 | - | 0.00 |
| BI167107 active | 0.59 | 0.53 | 1.00 |
|  | 0.65 | 0.59 | 1.00 |
|  | 0.70 | 0.68 | 1.00 |
|  | 1.00 | 1.00 | 0.47 |

***SI Table 14*** *GRIM results for the agonists-antagonist dataset from docking data obtained using crystallographic structures. The standard definition of hydrophobic contacts is applied. Thresholds discarding all docking poses are indicated as -.*

| **Reference binding mode** | **Threshold** | **Precision** |  |  | **Recall** |  |  |
| --- | --- | --- | --- | --- | --- | --- | --- |
|  |  | **Avg** | **Max** | **Min** | **Avg** | **Max** | **Min** |
| Epinephrine | 0.59 | 0.54 | 0.58 | 0.53 | 0.99 | 1.00 | 0.95 |
|  | 0.65 | 0.63 | 0.88 | 0.50 | 0.51 | 0.84 | 0.05 |
|  | 0.70 | 0.76 | 1.00 | 0.00 | 0.17 | 0.47 | 0.00 |
|  | 1.00 | - | - | - | 0.00 | 0.00 | 0.00 |
|  | 0.59 | 0.53 | 0.54 | 0.53 | 1.00 | 1.00 | 1.00 |
| HBI | 0.65 | 0.61 | 0.70 | 0.54 | 1.00 | 1.00 | 0.95 |
|  | 0.70 | 0.70 | 0.83 | 0.63 | 0.95 | 1.00 | 0.84 |
|  | 1.00 | - | - | - | 0.00 | 0.00 | 0.00 |
| BI167107-A | 0.59 | 0.53 | 0.54 | 0.53 | 1.00 | 1.00 | 1.00 |
|  | 0.65 | 0.64 | 0.68 | 0.58 | 1.00 | 1.00 | 1.00 |
|  | 0.70 | 0.73 | 0.79 | 0.66 | 0.99 | 1.00 | 0.95 |
|  | 1.00 | 1.00 | 1.00 | - | 0.01 | 0.05 | 0.00 |
| BI167107-I | 0.59 | 0.54 | 0.54 | 0.53 | 1.00 | 1.00 | 1.00 |
|  | 0.65 | 0.64 | 0.66 | 0.61 | 1.00 | 1.00 | 1.00 |
|  | 0.70 | 0.672 | 0.72 | 0.62 | 0.90 | 0.95 | 0.84 |
|  | 1.00 | - | - | - | 0.00 | 0.00 | 0.00 |

***SI Table 15*** *Average* *GRIM results on the agonists-antagonist dataset* from docking data obtained using the representative structures*. The Newhyd definition of hydrophobic contacts is applied.* The average value/highest value/lowest value are reported for each set of parameters. *Thresholds discarding all docking poses are indicated as -.*

| **Reference binding mode** | **Threshold** | **Precision** | **Recall** |
| --- | --- | --- | --- |
| Epinephrine | 0.59 | 0.53 | 1.00 |
|  | 0.65 | 0.68 | 1.00 |
|  | 0.70 | 0.83 | 1.00 |
|  | 1.00 | - | 0.00 |
|  | 0.59 | 0.54 | 1.00 |
| HBI | 0.65 | 0.59 | 1.00 |
|  | 0.70 | 0.75 | 0.95 |
|  | 1.00 | - | 0.00 |
| BI167107-A | 0.59 | 0.53 | 1.00 |
|  | 0.65 | 0.61 | 1.00 |
|  | 0.70 | 0.76 | 1.00 |
|  | 1.00 | - | 0.00 |

***SI Table 16*** *GRIM results on the agonists-antagonist dataset from docking data obtained using crystallographic structures. The Newhyd definition of hydrophobic contacts is applied. Thresholds discarding all docking poses are indicated as -.*

| **Reference binding mode** | **Threshold** | **Precision** | | | **Recall** | | |
| --- | --- | --- | --- | --- | --- | --- | --- |
|  |  | **Avg** | **Max** | **Min** | **Avg** | **Max** | **Min** |
| Epinephrine | 0.59 | 0.40 | 0.43 | 0.37 | 0.96 | 1.00 | 0.90 |
|  | 0.65 | 0.57 | 1.00 | 0.20 | 0.65 | 0.90 | 0.10 |
|  | 0.70 | 0.55 | 0.88 | 0.00 | 0.33 | 0.70 | 0.00 |
|  | 1.00 | - | - | - | 0.00 | 0.00 | 0.00 |
|  | 0.59 | 0.38 | 0.40 | 0.37 | 1.00 | 1.00 | 1.00 |
| HBI | 0.65 | 0.43 | 0.48 | 0.37 | 0.98 | 1.00 | 0.90 |
|  | 0.70 | 0.49 | 0.56 | 0.43 | 0.90 | 0.90 | 0.90 |
|  | 1.00 | - | - | - | 0.00 | 0.00 | 0.00 |
| BI167107 active | 0.59 | 0.39 | 0.43 | 0.37 | 0.98 | 1.00 | 0.90 |
|  | 0.65 | 0.46 | 0.50 | - | 0.75 | 1.00 | 0.00 |
|  | 0.70 | 0.57 | 0.59 | - | 0.75 | 1.00 | 0.00 |
|  | 1.00 | 0.00 | - | 0.00 | 0.00 | 0.00 | 0.00 |
| BI167107 inactive | 0.59 | 0.44 | 0.45 | 0.42 | 1.00 | 1.00 | 1.00 |
|  | 0.65 | 0.66 | 0.80 | 0.53 | 0.90 | 1.00 | 0.80 |
|  | 0.70 | 0.88 | 1.00 | 0.77 | 0.65 | 1.00 | 0.30 |
|  | 1.00 | - | - | - | 0.00 | 0.00 | 0.00 |

***SI Table 17*** *Average GRIM results on the agonists-inactives dataset* from docking data obtained using the representative structures*. The default definition of hydrophobic contacts is applied.* The average value/highest value/lowest value are reported for each set of parameters. *Thresholds discarding all docking poses are indicated as -.*

| **Reference binding mode** | **Threshold** | **Precision** | **Recall** |
| --- | --- | --- | --- |
| Epinephrine | 0.59 | 0.37 | 1.00 |
|  | 0.65 | 0.43 | 0.90 |
|  | 0.70 | 0.60 | 0.90 |
|  | 1.00 | - | 0.00 |
|  | 0.59 | 0.40 | 1.00 |
| HBI | 0.65 | 0.50 | 1.00 |
|  | 0.70 | 0.50 | 0.90 |
|  | 1.00 | - | 0.00 |
| BI167107 active | 0.59 | 0.38 | 1.00 |
|  | 0.65 | 0.48 | 1.00 |
|  | 0.70 | 0.56 | 0.90 |
|  | 1.00 | - | 0.00 |

***SI Table 18*** *GRIM results on the agonists-inactives dataset from docking data obtained using crystallographic structures. The default definition of hydrophobic contacts is applied. Thresholds discarding all docking poses are indicated as -.*

| **Ligand** | **Threshold** | **Precision** | | | **Recall** | | |
| --- | --- | --- | --- | --- | --- | --- | --- |
|  |  | **Avg** | **Max** | **Min** | **Avg** | **Max** | **Min** |
| Epinephrine | 0.59 | 0.39 | 0.42 | 0.36 | 0.99 | 1.00 | 0.90 |
|  | 0.65 | 0.51 | 0.64 | 0.29 | 0.70 | 0.90 | 0.20 |
|  | 0.70 | 0.31 | 0.75 | 0.00 | 0.21 | 0.80 | 0.00 |
|  | 1.00 | - | - | - | 0.00 | 0.00 | 0.00 |
|  | 0.59 | 0.39 | 0.40 | 0.37 | 1.00 | 1.00 | 1.00 |
| HBI | 0.65 | 0.46 | 0.50 | 0.43 | 0.97 | 1.00 | 0.90 |
|  | 0.70 | 0.55 | 0.62 | 0.47 | 0.86 | 1.00 | 0.50 |
|  | 1.00 | - | - | - | 0.00 | 0.00 | 0.00 |
| BI167107 active | 0.59 | 0.39 | 0.40 | 0.38 | 1.00 | 1.00 | 1.00 |
|  | 0.65 | 0.48 | 0.53 | 0.43 | 0.95 | 1.00 | 0.90 |
|  | 0.70 | 0.60 | 0.67 | 0.53 | 0.82 | 0.90 | 0.80 |
|  | 1.00 | - | - | - | 0.00 | 0.00 | 0.00 |
| BI167107 inactive | 0.59 | 0.40 | 0.43 | 0.38 | 1.00 | 1.00 | 1.00 |
|  | 0.65 | 0.50 | 0.50 | 0.50 | 1.00 | 1.00 | 1.00 |
|  | 0.70 | 0.71 | 0.82 | 0.60 | 0.90 | 0.90 | 0.90 |
|  | 1.00 | - | - | - | 0.00 | 0.00 | 0.00 |

***SI Table 19*** *GRIM results on the agonists-inactives dataset* from docking data obtained using the representative structures*. The Newhyd definition of hydrophobic contacts is applied.* The average value/highest value/lowest value are reported for each set of parameters. *Thresholds discarding all docking poses do are indicated as -.*

| **Ligand** | **Threshold** | **Precision** | **Recall** |
| --- | --- | --- | --- |
| Epinephrine | 0.59 | 0.40 | 1.00 |
|  | 0.65 | 0.50 | 0.90 |
|  | 0.70 | 0.53 | 0.80 |
|  | 1.00 | - | 0.00 |
|  | 0.59 | 0.40 | 1.00 |
| HBI | 0.65 | 0.53 | 1.00 |
|  | 0.70 | 0.56 | 0.90 |
|  | 1.00 | - | 0.00 |
| BI167107 active | 0.59 | 0.37 | 1.00 |
|  | 0.65 | 0.47 | 1.00 |
|  | 0.70 | 0.69 | 0.90 |
|  | 1.00 | - | 0.00 |

***SI Table 20*** *GRIM results on the agonists-inactives dataset from docking data obtained using crystallographic structures. The Newhyd definition of hydrophobic contacts is applied. Thresholds discarding all docking poses do are indicated as -.*

| **Ligand** | **Model** | **Threshold** | | | **Precision** | | | **Recall** | | |
| --- | --- | --- | --- | --- | --- | --- | --- | --- | --- | --- |
|  |  | **Avg** | **Max** | **Min** | **Avg** | **Max** | **Min** | **Avg** | **Max** | **Min** |
| Epinephrine | GRIM | 0.63 | 0.68 | 0.58 | 0.66 | 1.00 | 0.53 | 0.93 | 1.00 | 0.68 |
|  | GRIM - Newhyd | 0.63 | 0.68 | 0.60 | 0.66 | 1.00 | 0.53 | 0.90 | 1.00 | 0.68 |
|  | IFP | 0.38 | 0.67 | 0.13 | 0.64 | 1.00 | 0.53 | 0.93 | 1.00 | 0.74 |
|  | IFP - Polar | 0.48 | 1.00 | 0.25 | 0.65 | 1.00 | 0.53 | 0.94 | 1.00 | 0.74 |
|  | FitValue | 0.59 | 1.90 | 0.00 | 0.61 | 0.72 | 0.55 | 0.92 | 1.00 | 0.81 |
|  | Pharmtype | 1.43 | 4.00 | 0.00 | 0.60 | 0.75 | 0.55 | 0.92 | 1.00 | 0.71 |
| HBI | GRIM | 0.76 | 0.88 | 0.70 | 0.82 | 1.00 | 0.70 | 0.91 | 1.00 | 0.79 |
|  | GRIM - Newhyd | 0.75 | 0.80 | 0.70 | 0.81 | 0.94 | 0.65 | 0.91 | 1.00 | 0.79 |
|  | IFP | 0.58 | 0.69 | 0.44 | 0.76 | 1.00 | 0.56 | 0.89 | 1.00 | 0.63 |
|  | IFP - Polar | 0.57 | 0.67 | 0.44 | 0.71 | 0.89 | 0.56 | 0.92 | 1.00 | 0.84 |
|  | FitValue | 2.00 | 2.48 | 1.67 | 0.87 | 1.00 | 0.78 | 0.80 | 0.80 | 0.71 |
|  | Pharmtype | 4.00 | 5.00 | 3.00 | 0.79 | 0.94 | 0.63 | 0.80 | 1.00 | 0.71 |
| BI167107 active | GRIM | 0.82 | 0.91 | 0.76 | 0.91 | 1.00 | 0.81 | 0.92 | 1.00 | 0.89 |
|  | GRIM - Newhyd | 0.77 | 0.78 | 0.76 | 0.89 | 0.94 | 0.81 | 0.92 | 1.00 | 0.89 |
|  | IFP | 0.60 | 0.67 | 0.50 | 0.79 | 0.89 | 0.70 | 0.86 | 0.95 | 0.74 |
|  | IFP - Polar | 0.58 | 0.62 | 0.50 | 0.80 | 0.89 | 0.71 | 0.89 | 0.95 | 0.84 |
|  | FitValue | 2.16 | 2.56 | 1.69 | 0.93 | 1.00 | 0.83 | 0.84 | 0.90 | 0.81 |
|  | Pharmtype | 4.00 | 4.00 | 4.00 | 0.76 | 0.79 | 0.73 | 0.88 | 0.90 | 0.81 |
| BI167107 inactive | GRIM | 0.73 | 0.83 | 0.65 | 0.75 | 0.89 | 0.62 | 0.95 | 1.00 | 0.89 |
|  | GRIM - Newhyd | 0.71 | 0.76 | 0.66 | 0.74 | 0.86 | 0.63 | 0.97 | 1.00 | 0.95 |
|  | IFP | 0.64 | 0.83 | 0.50 | 0.73 | 1.00 | 0.53 | 0.88 | 1.00 | 0.68 |
|  | IFP - Polar | 0.67 | 0.83 | 0.50 | 0.76 | 1.00 | 0.53 | 0.84 | 1.00 | 0.68 |
|  | FitValue | 1.52 | 2.53 | 0.50 | 0.74 | 0.90 | 0.58 | 0.93 | 1.00 | 0.86 |
|  | Pharmtype | 4.50 | 5.00 | 4.00 | 0.78 | 0.89 | 0.68 | 0.78 | 0.81 | 0.76 |

***SI Table 21*** *Average optimal scoring threshold for GRIM, IFP similarity, and 3D pharmacophore based on the agonist-antagonist dataset* from docking data obtained using the representative structures*.* The average value/highest value/lowest value are reported for each scoring method.

| **Descriptor** | **Epinephrine** | **HBI** | **BI167107-A** | **BI167107-I** |
| --- | --- | --- | --- | --- |
| Avg. nodes | 25 | 60 | 67 | 62 |
| Min. nodes | 0 | 38 | 43 | 37 |
| Max. nodes | 46 | 81 | 85 | 88 |
| Avg. nodes † | 22 | 50 | 55 | 48 |
| Min. nodes † | 0 | 25 | 30 | 19 |
| Max. nodes † | 44 | 76 | 74 | 77 |
| Max. edge (Å) | 14 | 21 | 20 | 20 |
| Max. edge † (Å) | 14 | 21 | 20 | 20 |
| Avg HBA-P | 3.0 | 4.5 | 4.8 | 3.6 |
| Avg HBA-L | 2.7 | 3.4 | 3.8 | 2.6 |
| Avg HBA-C | 3.4 | 5.1 | 5.3 | 4.1 |
| Avg HBD-P | 0.3 | 2.6 | 1.7 | 1.4 |
| Avg HBD-L | 0.3 | 2.0 | 1.6 | 1.4 |
| Avg HBD-C | 0.3 | 2.6 | 1.7 | 1.4 |
| Avg HYD-P | 3.8 | 12.2 | 15.6 | 15.0 |
| Avg HYD-L | 3.4 | 9.6 | 11.1 | 10.7 |
| Avg HYD-C | 4.1 | 13.1 | 17.0 | 16.5 |
| Avg HYD-P † | 2.7 | 8.5 | 10.5 | 9.9 |
| Avg HYD-L † | 2.4 | 7.3 | 8.6 | 7.7 |
| Avg HYD-C † | 2.9 | 9.1 | 11.5 | 11.2 |
| Avg ANI-P | 1.3 | 2.0 | 1.9 | 2.0 |
| Avg ANI-L | 0.9 | 1.0 | 1.0 | 1.0 |
| Avg ANI-C | 1.3 | 2.0 | 1.9 | 2.0 |
| Avg ARO-P | 0.1 | 0.03 | 0.1 | 0.1 |
| Avg ARO-L | 0.1 | 0.03 | 0.1 | 0.1 |
| Avg ARO-C | 0.1 | 0.03 | 0.1 | 0.1 |

***SI Table 22*** *Statistics on the IPAs observed in the four MD simulations. † indicates values obtained with the Newhyd definition of hydrophobic contacts. HBA: hydrogen bond with the protein acting as acceptor, HBD: hydrogen bonds with the protein acting as donor, HYD: hydrophobic contacts, ANI: ionic interactions with the protein acting as anion, ARO: aromatic interactions, P: IPA placed on the protein, C: IPA placed in the midpoint, L: IPA placed on the ligand.*

| **Descriptor** | **Agonist** | **Antagonist** | **Agonist §** | **Inactive** |
| --- | --- | --- | --- | --- |
| Avg. nodes | 51 | 57 | 42 | 40 |
| Min. nodes | 9 | 9 | 11 | 3 |
| Max. nodes | 105 | 100 | 71 | 76 |
| Avg. nodes † | 40 | 44 | 32 | 30 |
| Min. nodes † | 6 | 0 | 6 | 3 |
| Max. nodes † | 83 | 81 | 61 | 66 |
| Max. edge (Å) | 26 | 26 | 23 | 23 |
| Max. edge † (Å) | 25 | 26 | 23 | 25 |
| Avg HBA-P | 2.4 | 1.6 | 1.6 | 1.0 |
| Avg HBA-L | 2.3 | 1.6 | 1.6 | 1.0 |
| Avg HBA-C | 2.8 | 1.9 | 2.1 | 1.2 |
| Avg HBD-P | 0.8 | 0.3 | 0.3 | 0.3 |
| Avg HBD-L | 0.8 | 0.3 | 0.3 | 0.3 |
| Avg HBD-C | 0.8 | 0.3 | 0.3 | 0.4 |
| Avg HYD-P | 12.7 | 16.1 | 10.9 | 11.3 |
| Avg HYD-L | 9.4 | 11.9 | 7.2 | 7.7 |
| Avg HYD-C | 15.0 | 19.2 | 12.2 | 12.9 |
| Avg HYD-P † | 8.6 | 11.2 | 7.2 | 7.1 |
| Avg HYD-L † | 7.4 | 9.6 | 5.8 | 5.9 |
| Avg HYD-C † | 10.3 | 13.6 | 8.2 | 8.2 |
| Avg ANI-P | 0.9 | 0.6 | 0.9 | 0.6 |
| Avg ANI-L | 0.6 | 0.5 | 0.7 | 0.5 |
| Avg ANI-C | 0.9 | 0.6 | 0.9 | 0.6 |
| Avg ARO-P | 0.7 | 0.6 | 0.9 | 0.9 |
| Avg ARO-L | 0.6 | 0.6 | 0.8 | 0.8 |
| Avg ARO-C | 0.7 | 0.6 | 0.9 | 0.9 |

***SI Table 23*** *Statistics on the IPAs observed in the docking poses. † indicates values obtained with the Newhyd definition of hydrophobic contacts. § indicates agonist molecules from the agonist-inactive dataset. HBA: hydrogen bond with the protein acting as acceptor, HBD: hydrogen bonds with the protein acting as donor, HYD: hydrophobic contacts, ANI: ionic interactions with the protein acting as anion, ARO: aromatic interactions, P: IPA placed on the protein, C: IPA placed in the midpoint, L: IPA placed on the ligand*

REFERENCES

1. Gaulton A, Hersey A, Nowotka M, et al (2017) The ChEMBL database in 2017. Nucleic Acids Res 45:D945–D954. https://doi.org/10.1093/nar/gkw1074

2. Crooks CR, Wright J, Callery PS, Moreton JE (1979) Synthesis and preliminary biological studies of 4- and 5-[2-hydroxy-3-(isopropylamino)propoxy]benzimidazoles: selective β2 adrenergic blocking agents. J Med Chem 22:210–214. https://doi.org/10.1021/jm00188a019

3. Carre MC, Youlassani A, Caubere P (1984) Synthesis of a novel series of (aryloxy)propanolamines: new selective .beta.2-blocking agents. J Med Chem 27:792–799. https://doi.org/10.1021/jm00372a016

4. El Tayar N, Carrupt PA, Van de Waterbeemd H, Testa B (1988) Modeling of .beta.-adrenoceptors based on molecular electrostatic potential studies of agonists and antagonists. J Med Chem 31:2072–2081. https://doi.org/10.1021/jm00119a004

5. Altosaar K, Balaji P, Bond RA, et al (2021) Adrenoceptors in GtoPdb v.2021.3. GtoPdb CITE 2021:. https://doi.org/10.2218/gtopdb/F4/2021.3

6. Kooistra AJ, Mordalski S, Pándy-Szekeres G, et al (2021) GPCRdb in 2021: integrating GPCR sequence, structure and function. Nucleic Acids Research 49:D335–D343. https://doi.org/10.1093/nar/gkaa1080

7. Scharf MM, Bünemann M, Baker JG, Kolb P (2019) Comparative Docking to Distinct G Protein–Coupled Receptor Conformations Exclusively Yields Ligands with Agonist Efficacy. Mol Pharmacol 96:851–861. https://doi.org/10.1124/mol.119.117515
